# Supplementary material for: MENDER: fast and scalable tissue structure identification in spatial omics data
Source: Nat Commun. 2024 Jan 5;15:207. doi: 10.1038/s41467-023-44367-9 (PMC10770058; doi:10.1038/s41467-023-44367-9)
Supplement: Supplementary file 1 — Supplementary Information [file 41467_2023_44367_MOESM1_ESM.pdf]

Supplementary Figure 1

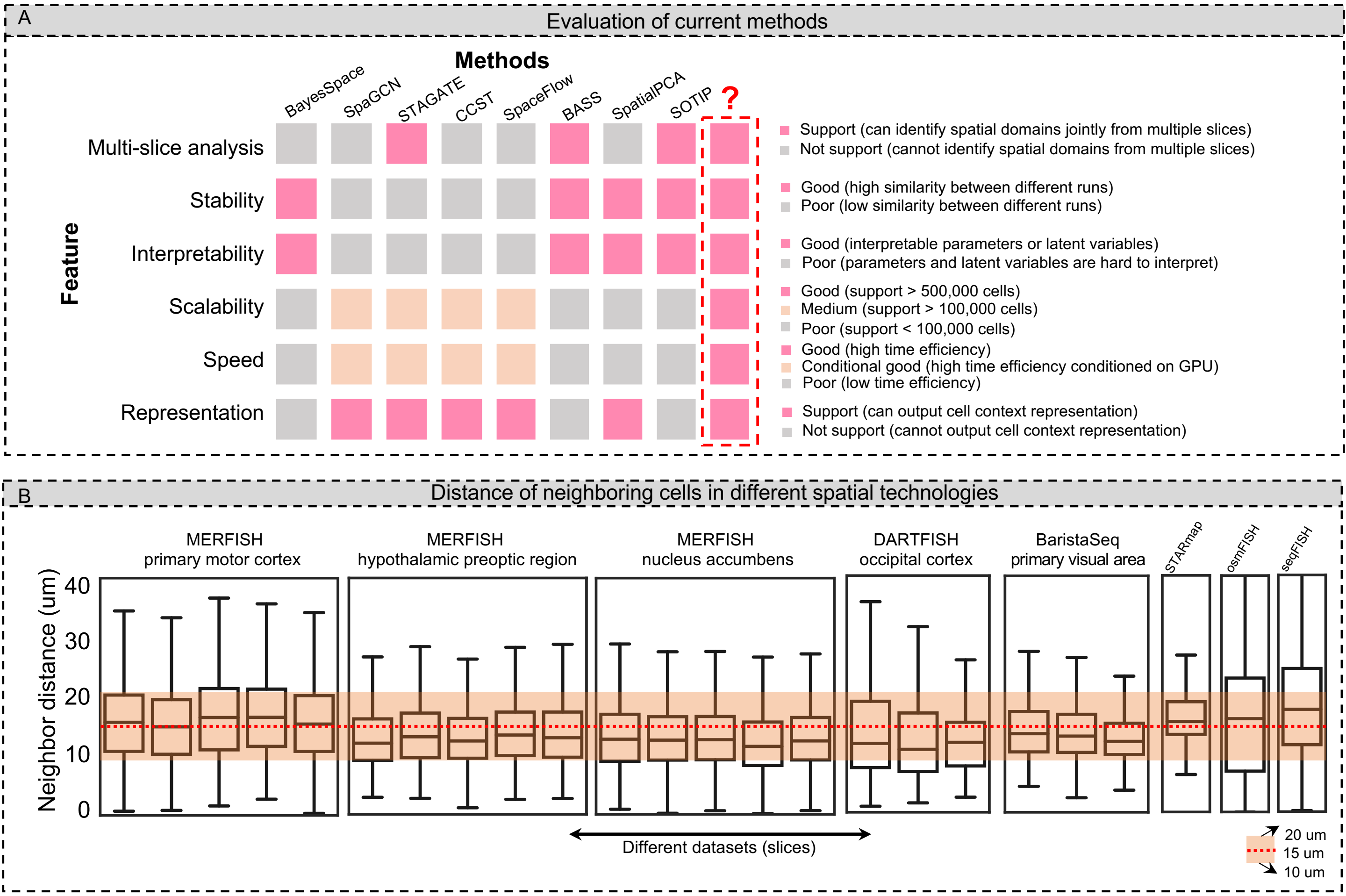

Supplementary Figure 1. Motivation of MENDER

A: Evaluation of state-of-the-art methods for spatial domain identification. Each row is a criterion and each column is a method. Colors indicate the performance. Detailed explanations can be found in “Methods”.

B: Distance of neighboring cells in different spatial technologies. Each box is the distance distribution of neighboring cells in a dataset. Distances between 10 um to 20 um are highlighted with orange and a distance of 15 um is indicated with the red dashed line.

Source data are provided as a Source Data file Source data are provided as a Source Data file.

Supplementary Figure 2

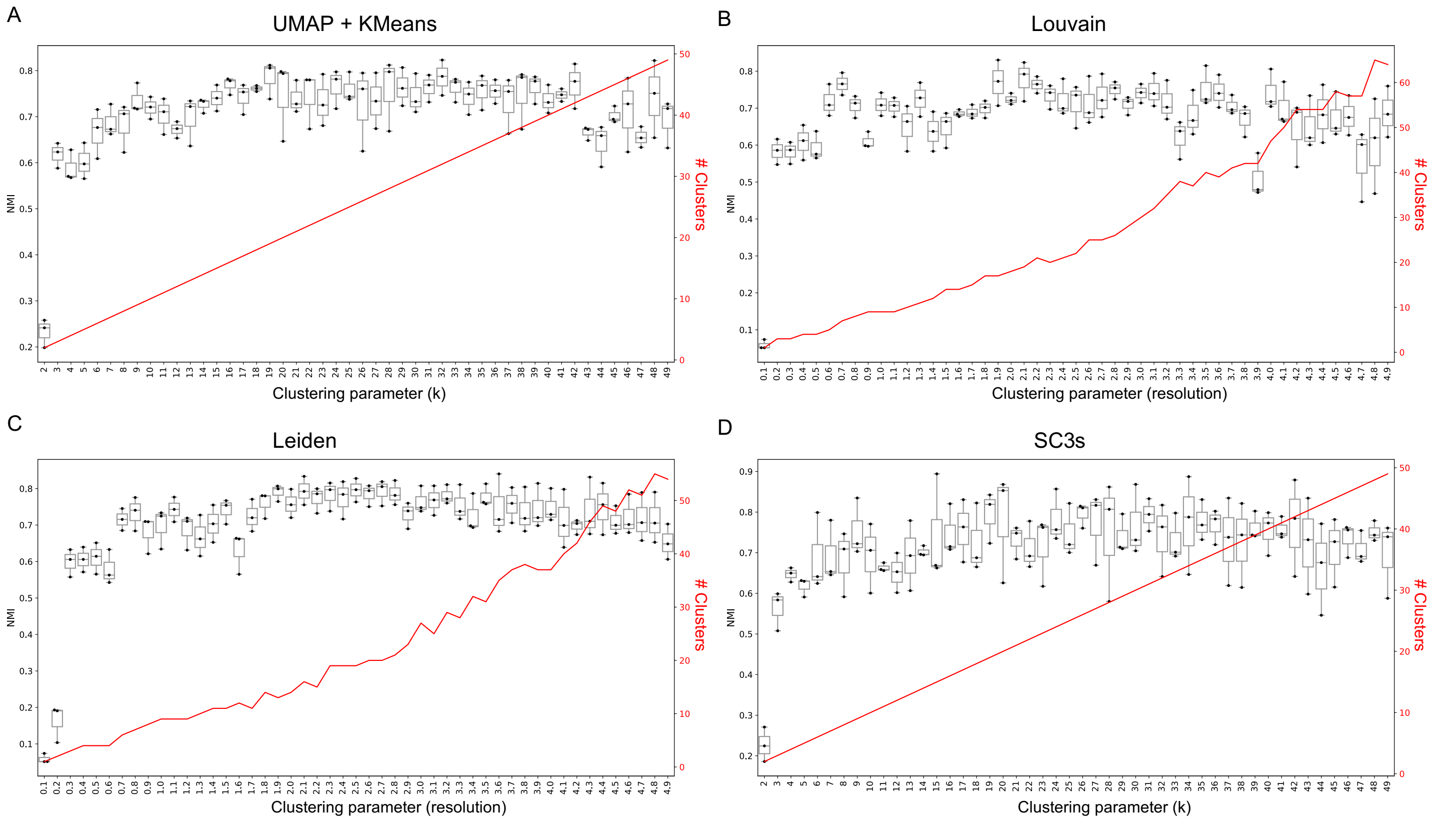

Supplementary Figure 2. Influence of cell clustering methods and parameters on MENDER performance using STARmap dataset
MENDER's pipeline incorporates a "Cell Group" step. In this figure, we assess the influence of different cell clustering methods on MENDER's performance using the STARmap dataset. Four distinct cell clustering methods were tested: (A) Kmeans on UMAP embedding (referred to as UMAP + KMeans), (B) Louvain, (C) Leiden, and (D) SC3s. In addition to evaluating the clustering methods themselves, we delved into how varying parameters within these methods affect MENDER's performance. For UMAP + KMeans (A) and SC3s (D), the defining parameter is 'k' (the anticipated number of clusters). Conversely, for Louvain (B) and Leiden (C), the defining parameter is 'resolution', which pertains to clustering granularity. As illustrated in (A), the plot demonstrates MENDER's performance (quantified by NMI) relative to clustering parameters (i.e., k) (depicted by black boxplots). Concurrently, the red line-plot shows the number of clusters in response to changes in clustering parameters. Source data are provided as a Source Data file Source data are provided as a Source Data file.

Supplementary Figure 3

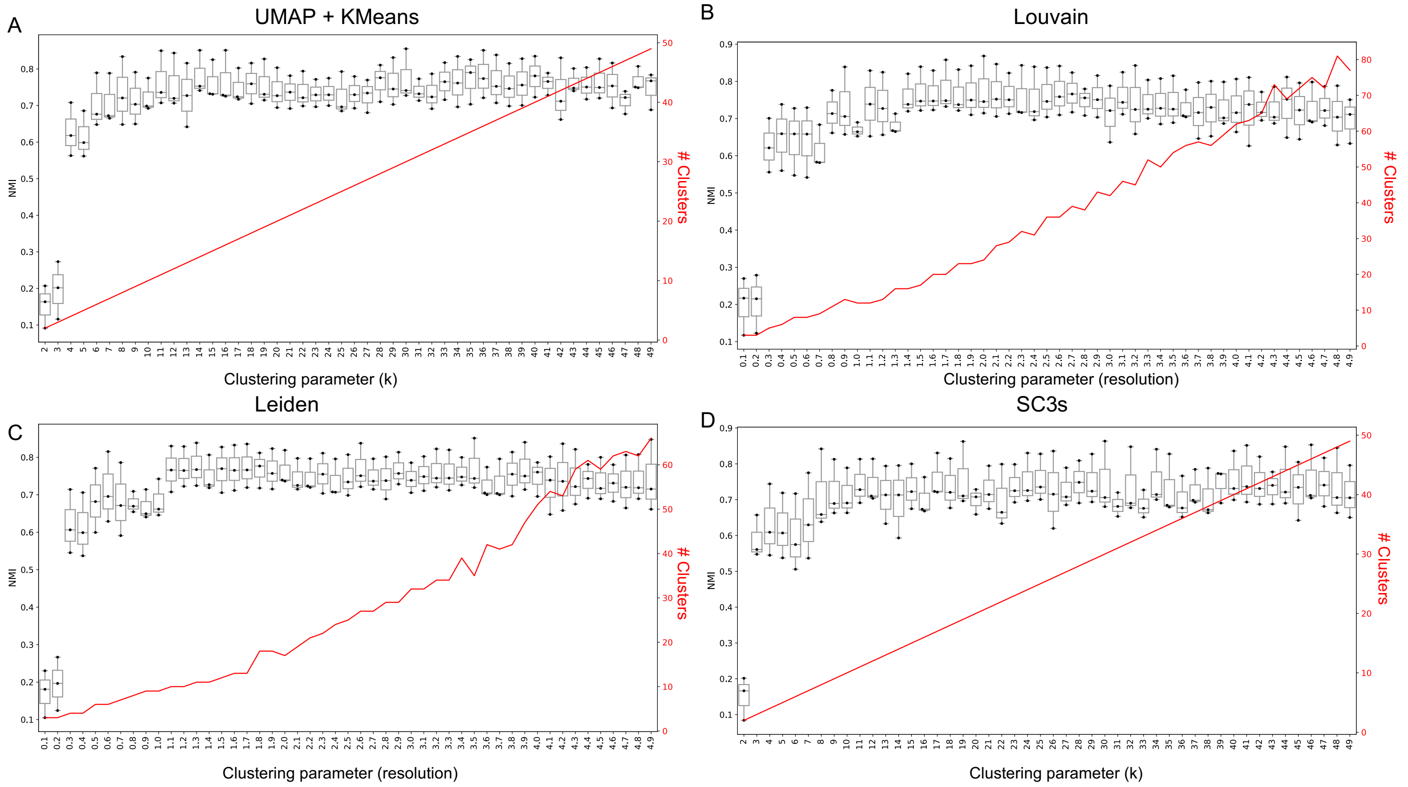

Supplementary Figure 3. Influence of cell clustering methods and parameters on MENDER performance using BaristaSeq dataset
MENDER's pipeline incorporates a "Cell Group" step. In this figure, we assess the influence of different cell clustering methods on MENDER's performance using the BaristaSeq dataset. Four distinct cell clustering methods were tested: (A) Kmeans on UMAP embedding (referred to as UMAP + KMeans), (B) Louvain, (C) Leiden, and (D) SC3s. In addition to evaluating the clustering methods themselves, we delved into how varying parameters within these methods affect MENDER's performance. For UMAP + KMeans (A) and SC3s (D), the defining parameter is 'k' (the anticipated number of clusters). Conversely, for Louvain (B) and Leiden (C), the defining parameter is 'resolution', which pertains to clustering granularity. As illustrated in (A), the plot demonstrates MENDER's performance (quantified by NMI) relative to clustering parameters (i.e., k) (depicted by black boxplots). Concurrently, the red line-plot shows the number of clusters in response to changes in clustering parameters. Source data are provided as a Source Data file Source data are provided as a Source Data file.

Supplementary Figure 4

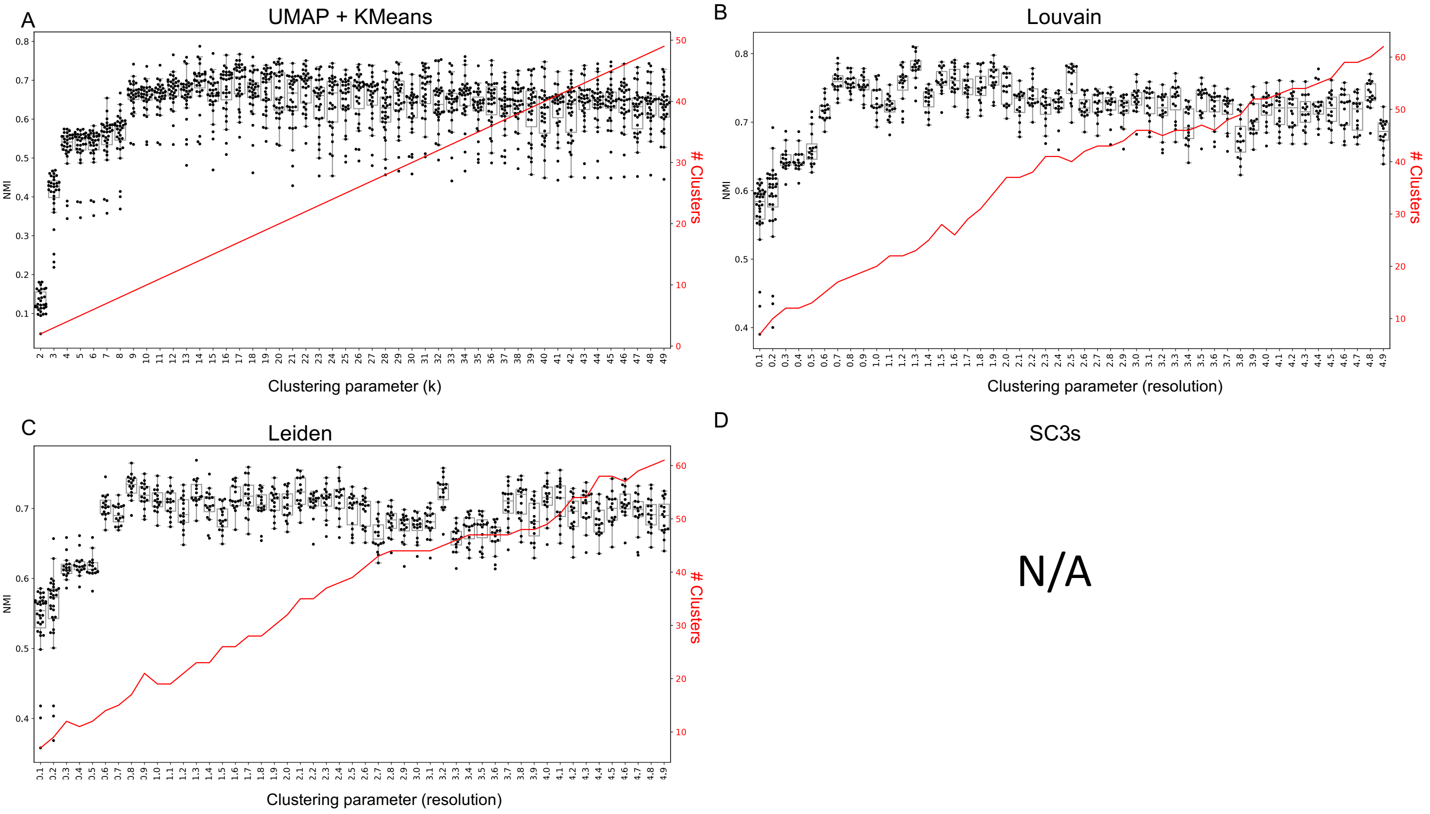

Supplementary Figure 4. Influence of cell clustering methods and parameters on MENDER performance using MERFISH dataset
MENDER's pipeline incorporates a "Cell Group" step. In this figure, we assess the influence of different cell clustering methods on MENDER's performance using the MERFISH dataset. Four distinct cell clustering methods were tested: (A) Kmeans on UMAP embedding (referred to as UMAP + KMeans), (B) Louvain, (C) Leiden, and (D) SC3s. In addition to evaluating the clustering methods themselves, we delved into how varying parameters within these methods affect MENDER's performance. For UMAP + KMeans (A) and SC3s (D), the defining parameter is 'k' (the anticipated number of clusters). Conversely, for Louvain (B) and Leiden (C), the defining parameter is 'resolution', which pertains to clustering granularity. As illustrated in (A), the plot demonstrates MENDER's performance (quantified by NMI) relative to clustering parameters (i.e., k) (depicted by black boxplots). Concurrently, the red line-plot shows the number of clusters in response to changes in clustering parameters.
Note that memory issues occurred when using SC3s as cell clustering method, so we labeled "N/A". Source data are provided as a Source Data file
Source data are provided as a Source Data file.

Supplementary Figure 5

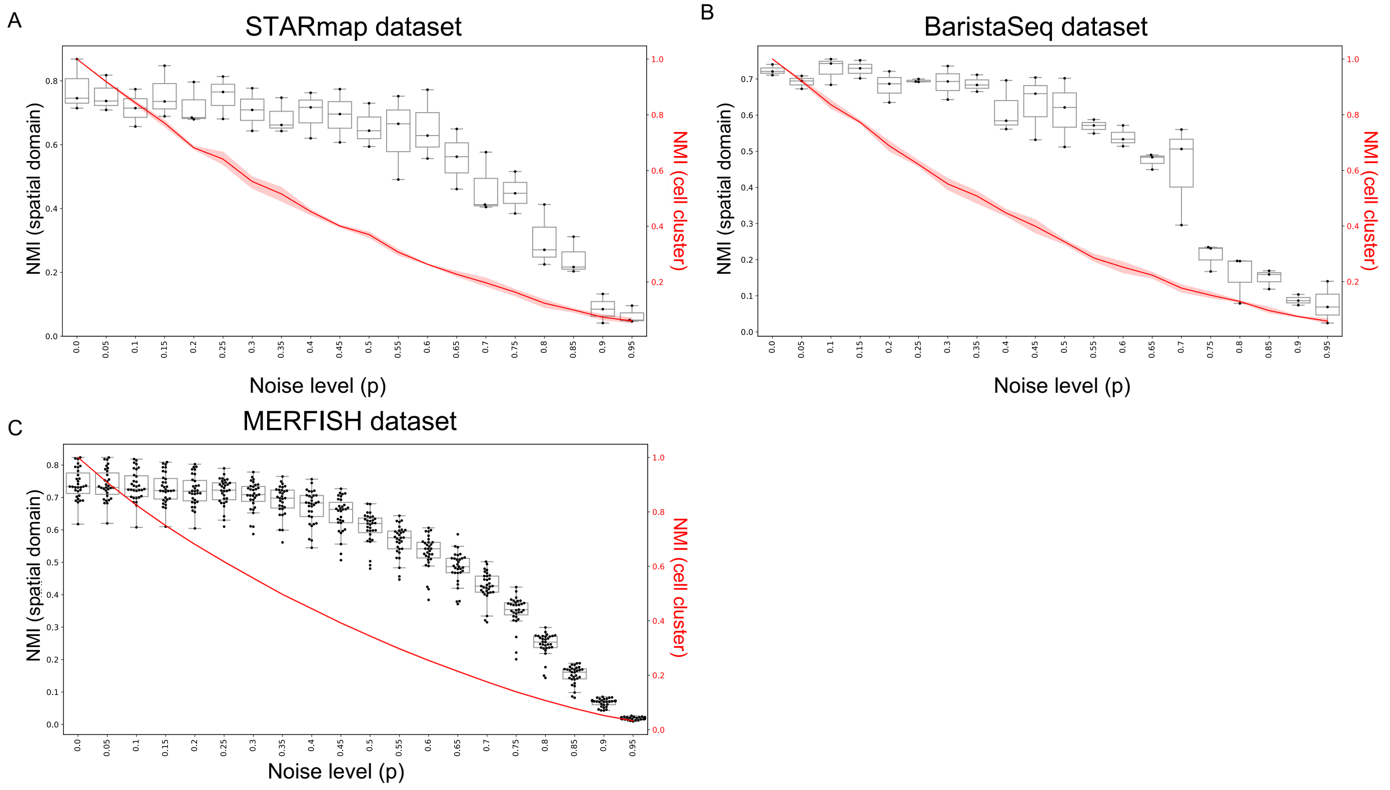

Supplementary Figure 5. Influence of noisy cell clusters on MENDER performance

We used three datasets to explore MENDER performance when different levels of noise of cell clusters exist. For this purpose, we introduced varying levels of noise into the cell group step, using datasets STARmap (A), BaristaSeq (B), and MERFISH (C). For each dataset, the black boxplots illustrate MENDER's performance (measured in terms of NMI) as a function of the introduced noise level. The line plot (in red) indicates the NMI between the noisy cell group label and the original cell group label, mapped against the noise level. By "original cell group label", we refer to MENDER's default cell grouping method, which is Leiden with a resolution of 2. The term "noisy cell group label" means that for each cell, the group label has a probability 1-p of being the original cell group label and a probability p of being a randomly chosen label from the original label set. Observations from plots (A-C) reveal that MENDER's performance experiences only a marginal decline when the noise level is below 0.5, underscoring MENDER's robustness to low-quality cell group labels. Source data are provided as a Source Data file Source data are provided as a Source Data file.

Supplementary Figure 6

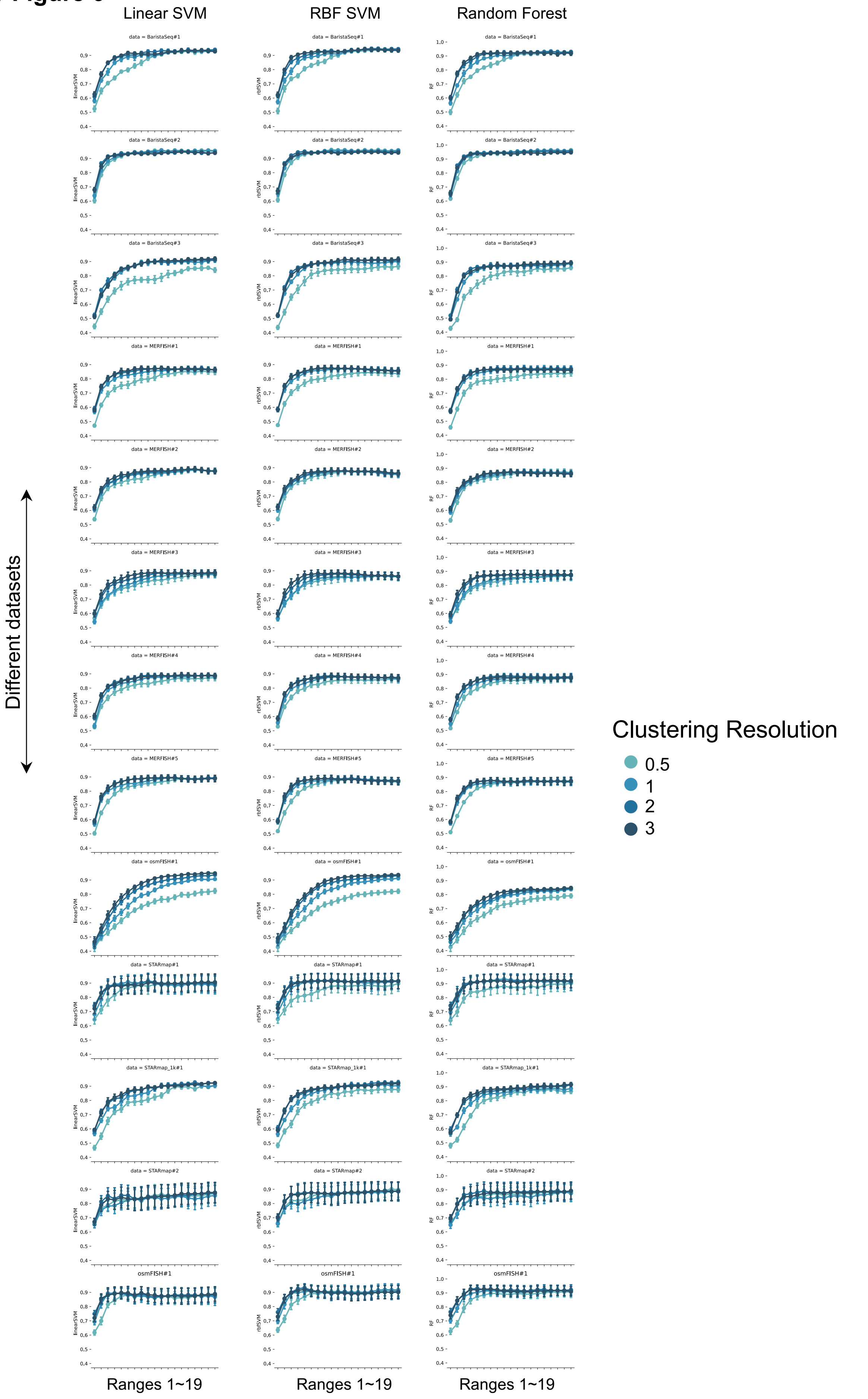

Supplementary Fig. 6. Representation power of MENDER with different parameters
The spatial domain prediction was performed on 13 spatial datasets (for each row) using 3 classifiers (i.e., Linear SVM, RBF SVM, and Random Forest). Each plot shows accuracy of the prediction (10-fold cross validation) as the function of the number of ranges. 4 Different clustering resolution (used for cell state clustering) were tested (represented by different point colors). Source data are provided as a Source Data file Source data are provided as a Source Data file.

Supplementary Figure 7

A

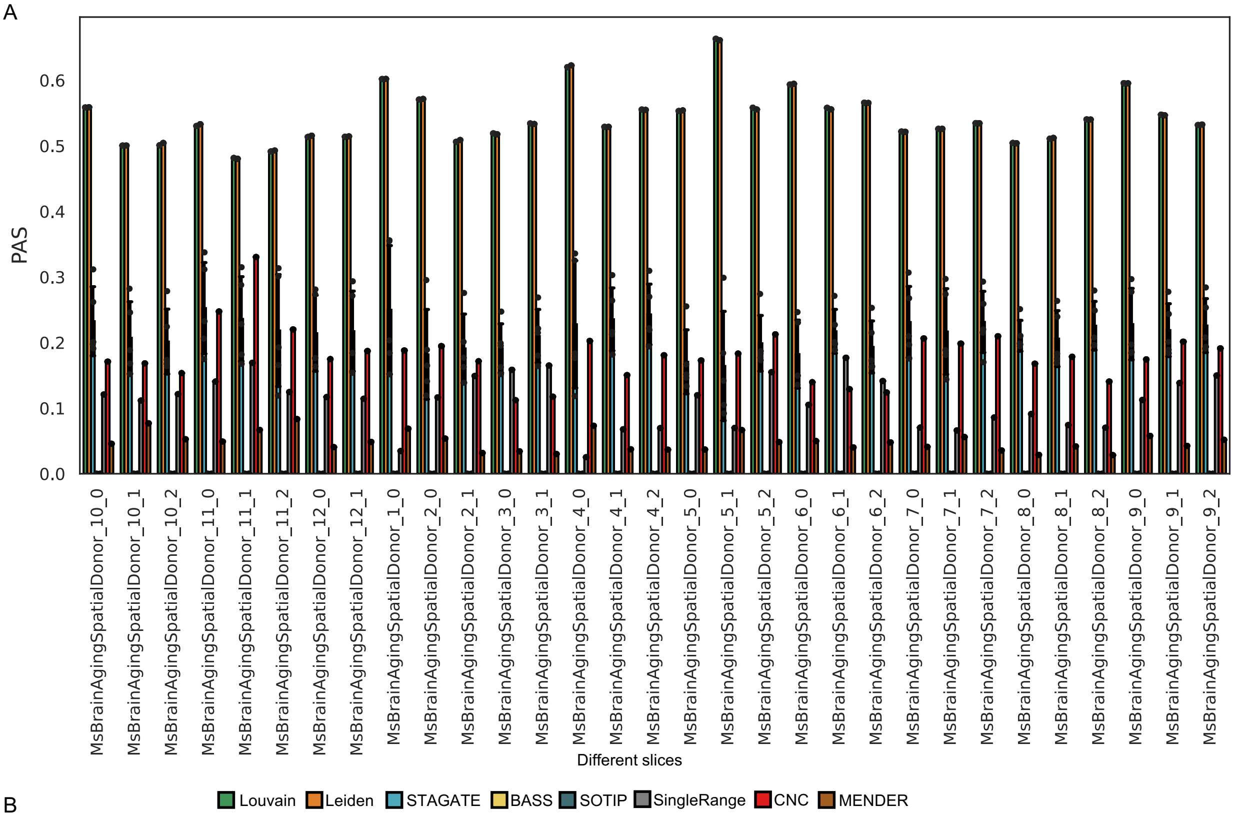

B

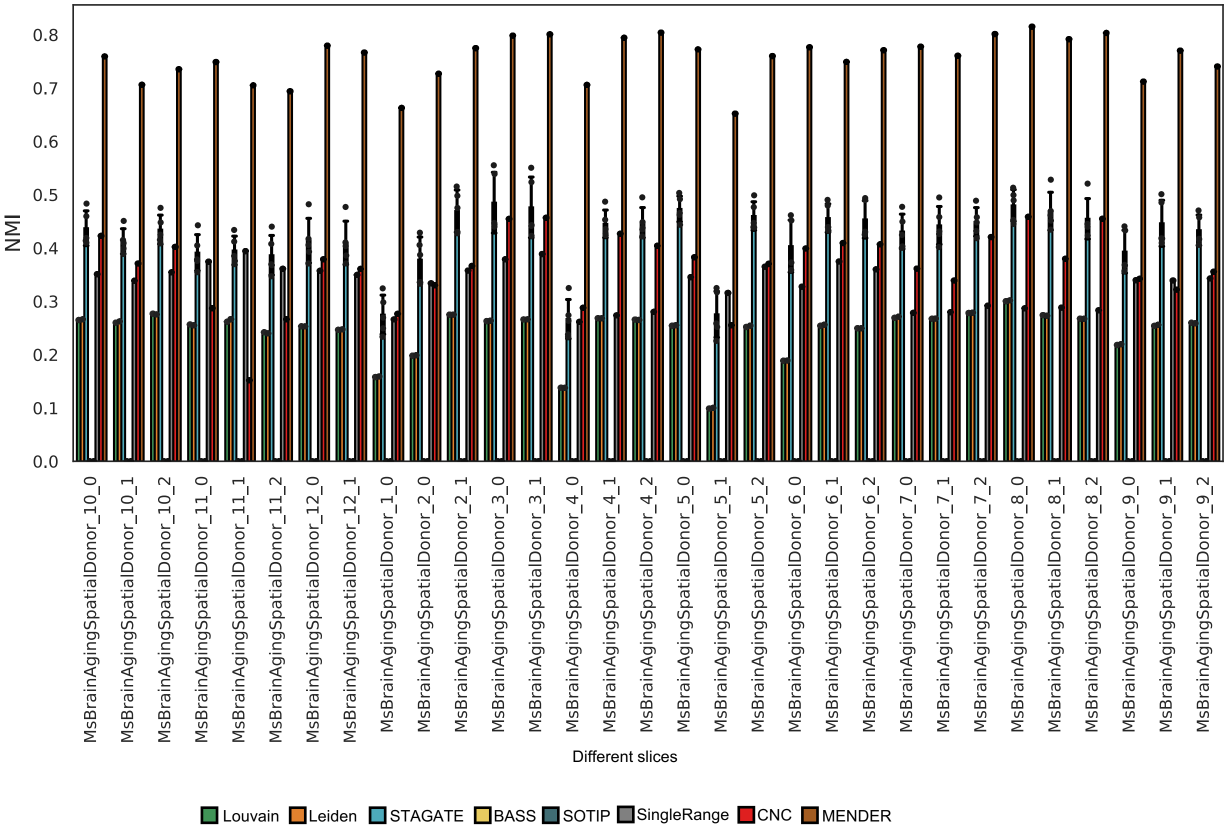

Supplementary Figure 7. Benchmarking analysis on MERFISH dataset.
The dataset is from the mouse frontal cortex area from 31 slices (Figure 3M-P). PAS (A) and NMI (B) are used to evaluate different methods for each slice. Source data are provided as a Source Data file Source data are provided as a Source Data file.

Supplementary Figure 8

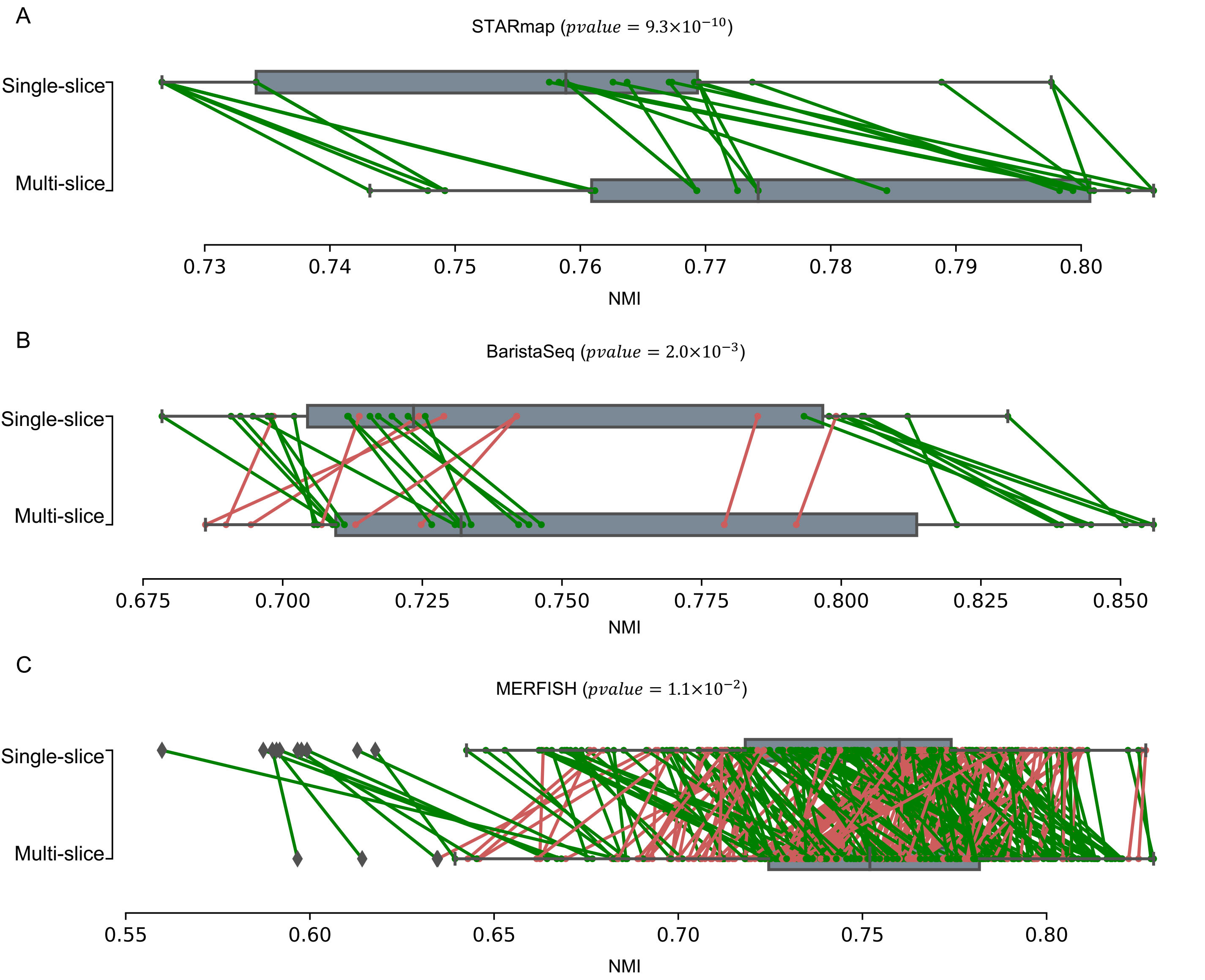

Supplementary Figure 8. Performance comparison between two versions of MENDER (multi-slice vs single-slice analysis)
This figure compares multi-slice and single-slice analysis using MENDER, applied to STARmap (A), BaristaSeq (B), and MERFISH (C) datasets. Each experiment was performed for 10 replicated runs. Therefore, the number of points for STARmap is 30, for BaristaSeq it's 30, and for MERFISH it's 310. P-values were computed using a one-sided Wilcoxon rank-sum test (multi-slice analyses are claimed to be higher). For each pair of experiments, the green line indicates improved NMI when comparing the multi-slice and single-slice versions of MENDER, while the red line indicates reduced NMI when comparing the multi-slice and single-slice versions of MENDER. Source data are provided as a Source Data file Source data are provided as a Source Data file.

Supplementary Figure 9

Spatial technology	Tissue	# Slices	Commercialization	Resolution	Parameter	Reproducible Notebook Name
STARmap	Prelimbic area	3	Stellaromics	Single-cell	Scale=6 | radius=15um | k=4	STARmap_PrelimbicArea
BaristaSeq	Visual cortex	3	No	Single-cell	Scale=6 | radius=15um | k=6	BaristaSeq_VISp
MERFISH	Frontal cortex	31	No	Single-cell	Scale=6 | radius=15um | k=8	MERFISH_aging
MERSCOPE	Brain	9	Vizgen	Single-cell	Scale=6 | radius=15um | res=1.5	MERSCOPE
Spatial Transcriptomics (ST)	Olfactory bulb	1	No	Spot	Scale=3 | nn=4 | res=0.5	ST_MOB
10X Visium	Brain	2	10X Genomics	Spot	Scale=3 | nn=6 | k=4	Visium_brain01
10X Visium	Olfactory bulb	1	10X Genomics	Spot	Scale=2 | nn=6 | k=4	Visium_MOB
Slide-seq	Cerebellum	1	No	Spot	Scale=4 | radius=15um | res=0.5	Slide-seq_Cerebellum
Slide-seq	Hippocampus	1	No	Spot	Scale=4 | radius=15um | res=0.5	Slide-seq_Hippocampus
Slide-seq	Olfactory bulb	1	No	Spot	Scale=4 | radius=15um | res=0.5	Slide-seq_MOB
Stereo-Seq	Olfactory bulb	1	BGI	Single-cell	Scale=6 | radius=15um | res=0.5	StereoSeq_MOB
osmFISH	Somatosensory cortex	1	No	Single-cell	Scale=6 | radius=15um | k=11	osmFISH_SS
ExSeq	Visual cortex	1	No	Single-cell	Scale=6 | radius=15um | res=0.5	ExSeq_VISp
STARmapPlus	Brain	8	Stellaromics	Single-cell	Scale=6 | radius=15um | res=0.5	STARmapPlus_AD

Supplementary Figure 9. Data information.
The specific details of the spatial datasets tested in the manuscript. The figure offers an overview of various attributes including the type of spatial technologies used, the tissue samples, the number of slices, and whether these technologies are commercialized. Importantly, it also highlights the spatial resolution details, the specific parameters employed when implementing MENDER, and the corresponding Jupyter notebook name for each dataset, which can be found the online webpage, https://mender-tutorial.readthedocs.io/en/latest/. For parameters, 'k' is the expected number of domains, and 'res' is the clustering resolution. When the number of domains is known, 'k' can be set. Otherwise, 'res=0.5' (default value) is first tested, and then the user can assess the visualization result to adjust 'res' according to their needs.

Supplementary Figure 10

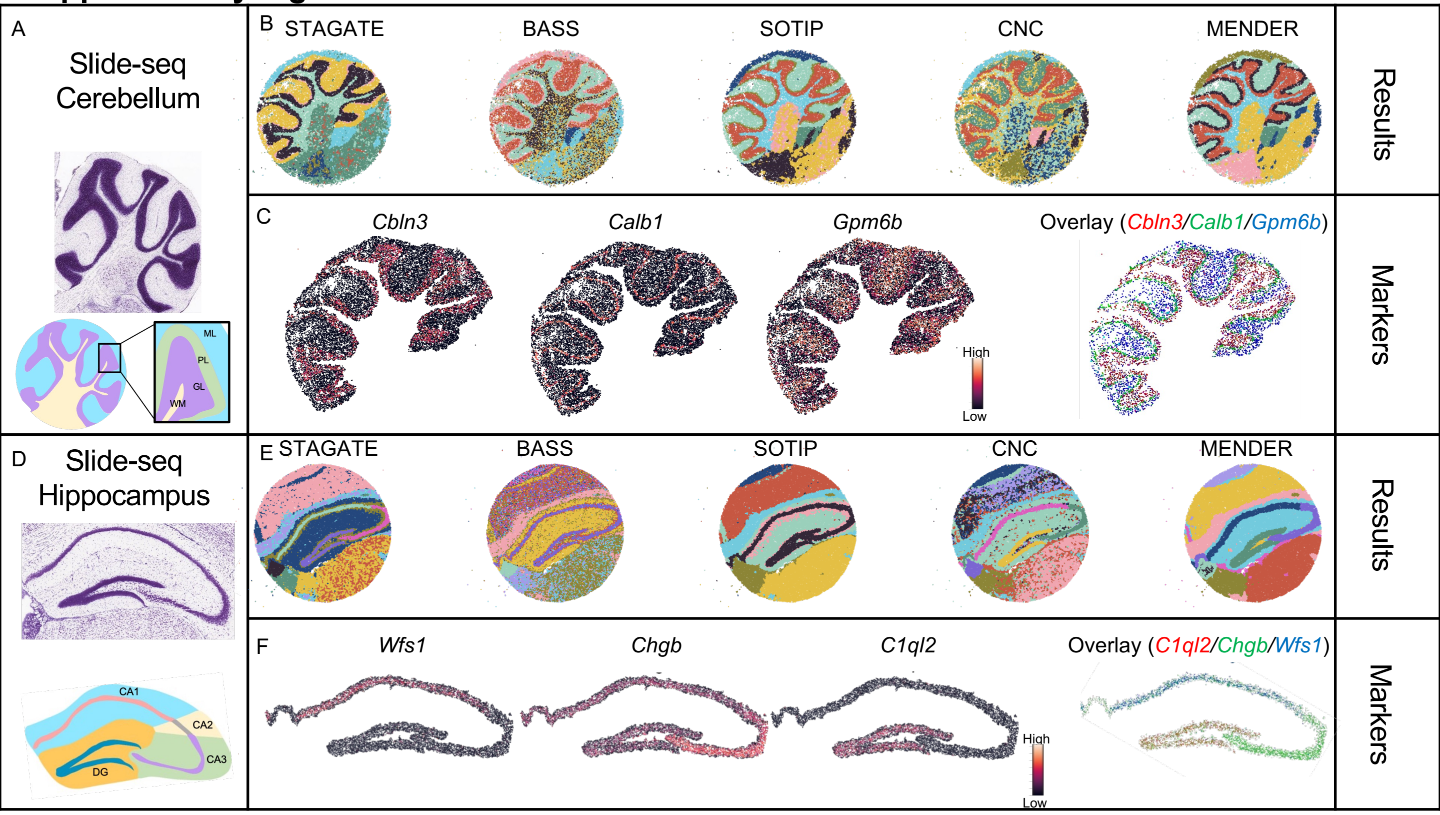

Supplementary Figure 10. Methods comparison on extended data types.
STAGATE, BASS, SOTIP, CNC, and MENDER were compared on two slide-seq datasets: Cerebellum (A-C) and Hippocampus (D-F). For the cerebellum data, the structure reference is shown in (A), the results of different methods are shown in (B), and structural markers are shown in (C). The same applies to (D, E, F). ML: Molecular Layer. GL: Granule layer. PL: Purkinje Layer. WM: White Matter. CA: Cornu Ammonis. DG: Dentate Gyrus. Note that (F) has been rotated for visualization purpose. Source data are provided as a Source Data file Source data are provided as a Source Data file.

Supplementary Figure 11

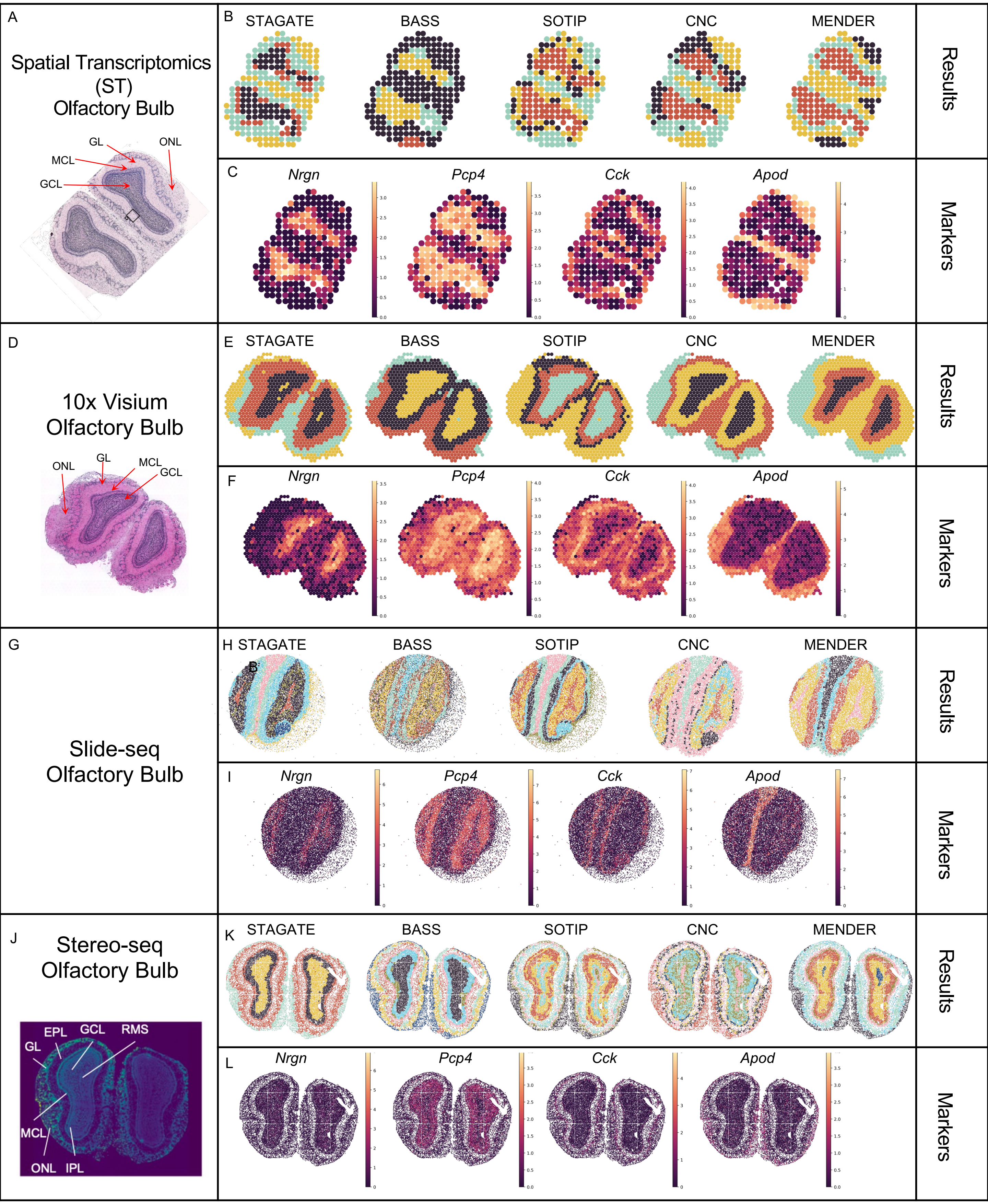

Supplementary Figure 11. Methods comparison on extended data types.
STAGATE, BASS, SOTIP, CNC, and MENDER were compared on mouse olfactory bulb (MOB) data obtained from four different spatial technologies, including Spatial Transcriptomics (A-C), 10x Visium (D-F), Slide-seq (G-I), and Stereo-seq (J-L). For each experiment, the histological image was sourced from the original publication for tissue structure reference (except for Slide-seq data, as we couldn't find the paired histology image in the original paper). Structural markers of the MOB for each experiment were also visualized (C, F, I, L). GCL: Granule Cell Layer. IPL: Internal Plexiform Layer. MCL: Mitral Cell Layer. EPL: External Plexiform Layer. ONL: Olfactory Nerve Layer. RMS: Rostral Migratory Stream. GL: Glomerular Layer. Source data are provided as a Source Data file Source data are provided as a Source Data file.

Supplementary Figure 12

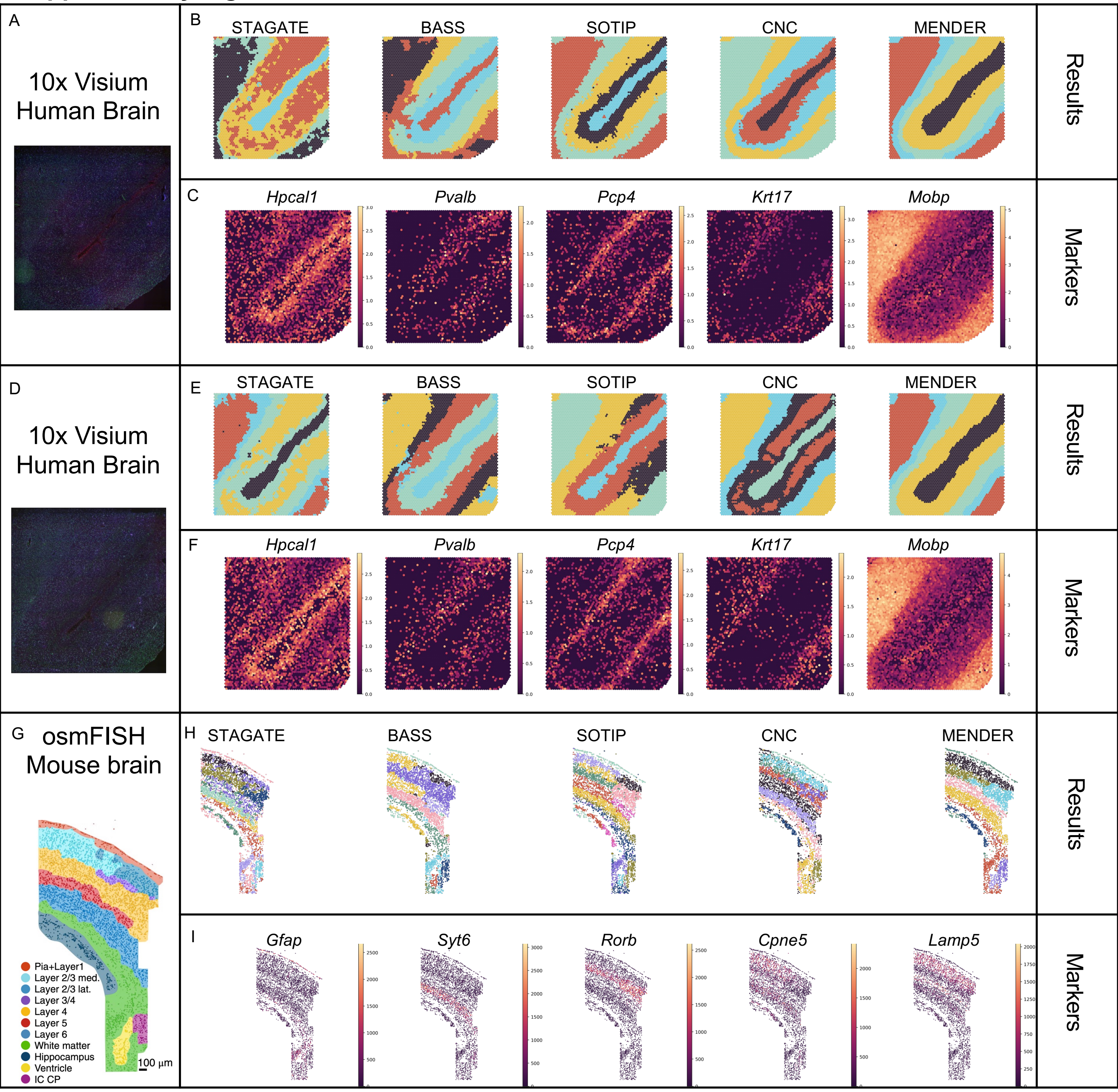

Supplementary Figure 12. Methods comparison on extended data types.
STAGATE, BASS, SOTIP, CNC, and MENDER were compared on brain cortex tissue data obtained from two different spatial technologies: 10x Visium (A-F) and osmFISH (G-I). For the 10x Visium data, the histological image was provided (A, D). For the osmFISH data, the tissue anatomy annotation was sourced from the original publication (G). Source data are provided as a Source Data file Source data are provided as a Source Data file.

Supplementary Figure 13

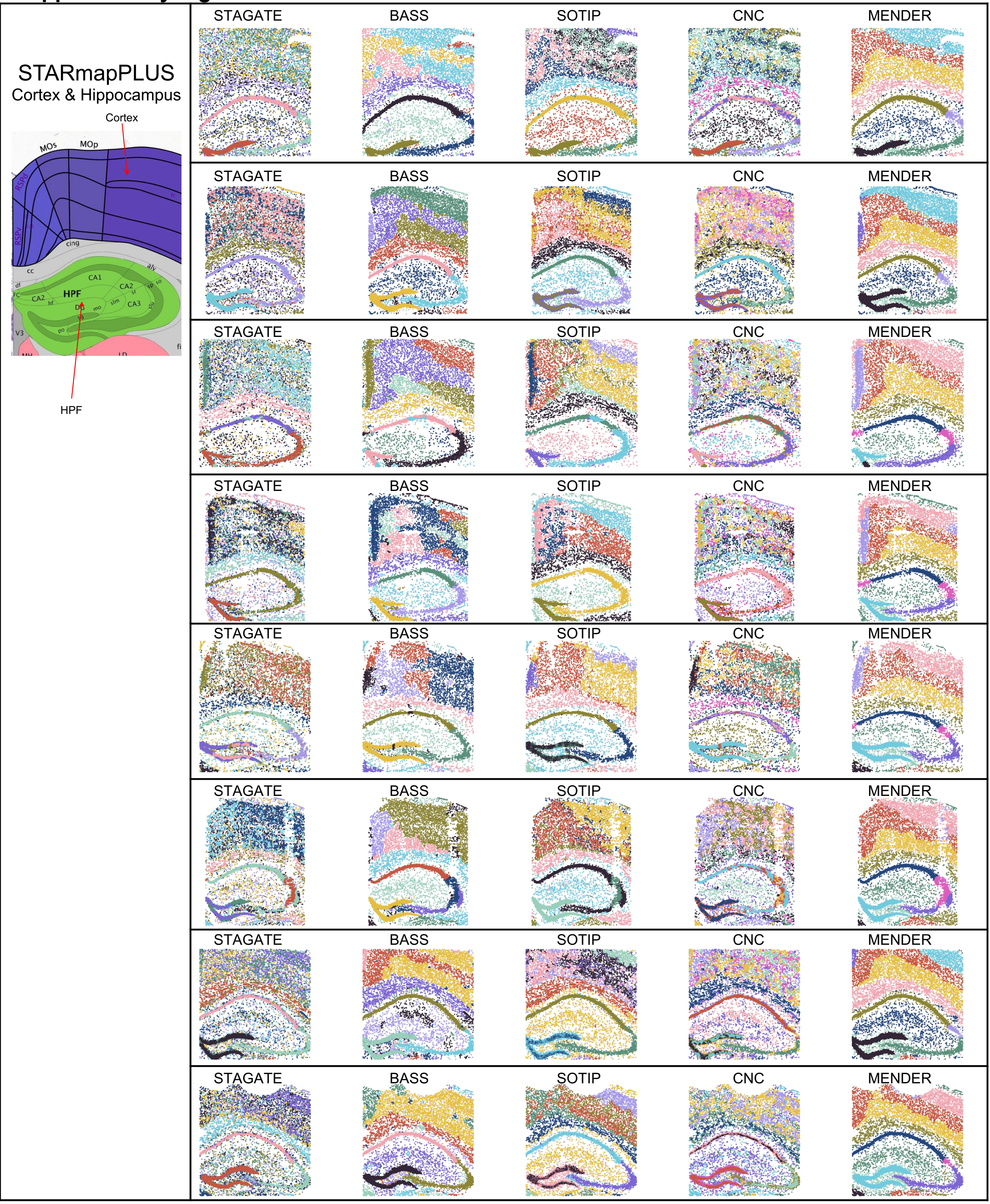

Supplementary Figure 13. Methods comparison on extended data types.
STAGATE, BASS, SOTIP, CNC, and MENDER were compared on STARmapPLUS datasets containing 8 samples. Each row displays results for each sample (8 rows in total). The Allen reference atlas was provided for comparison with the results. HPF: Hippocampal Formation. Source data are provided as a Source Data file Source data are provided as a Source Data file.

Supplementary Figure 14

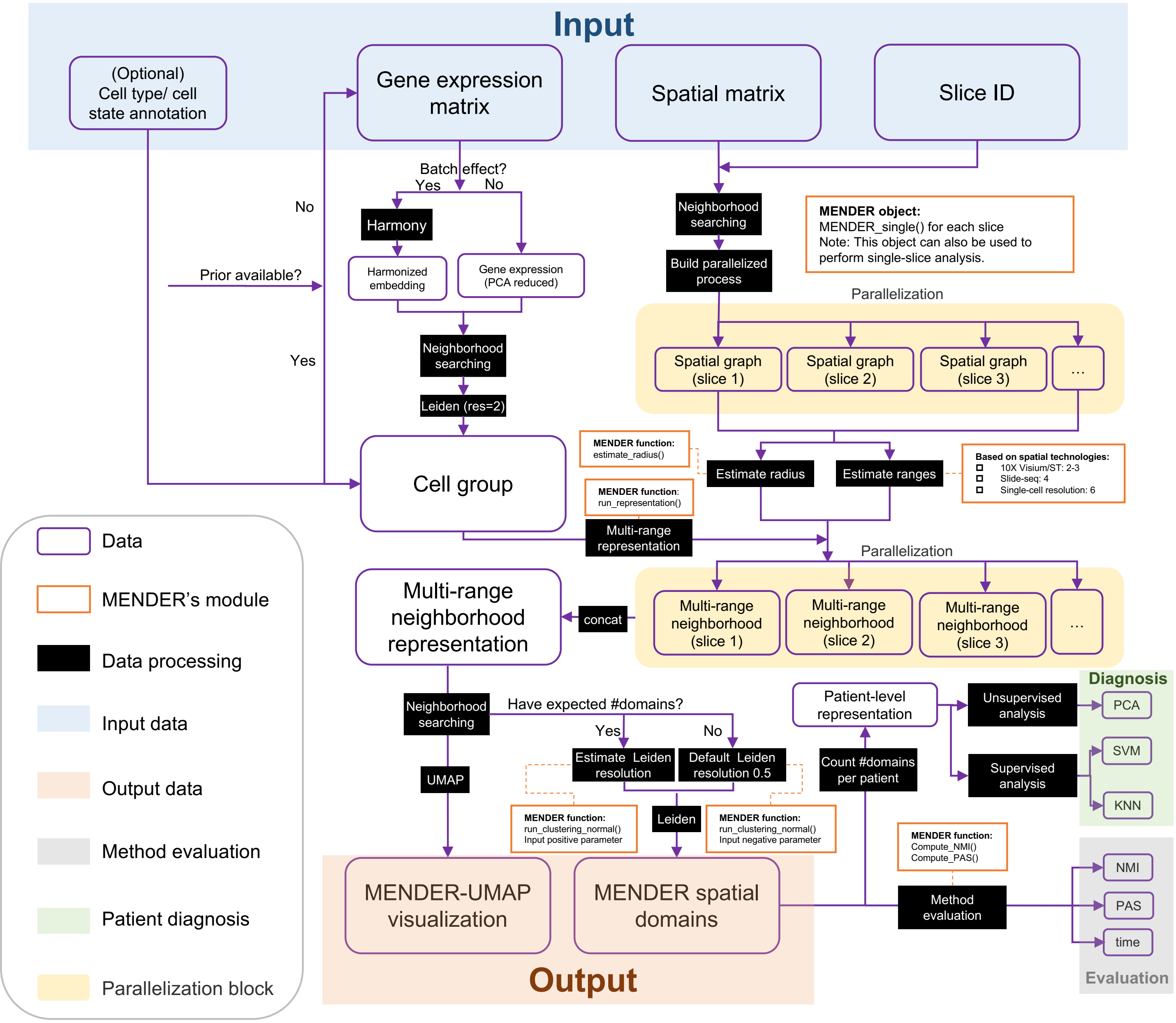

Supplementary Figure 14. MENDER's pipeline, a detailed version of Figure 1.

Supplementary Figure 15

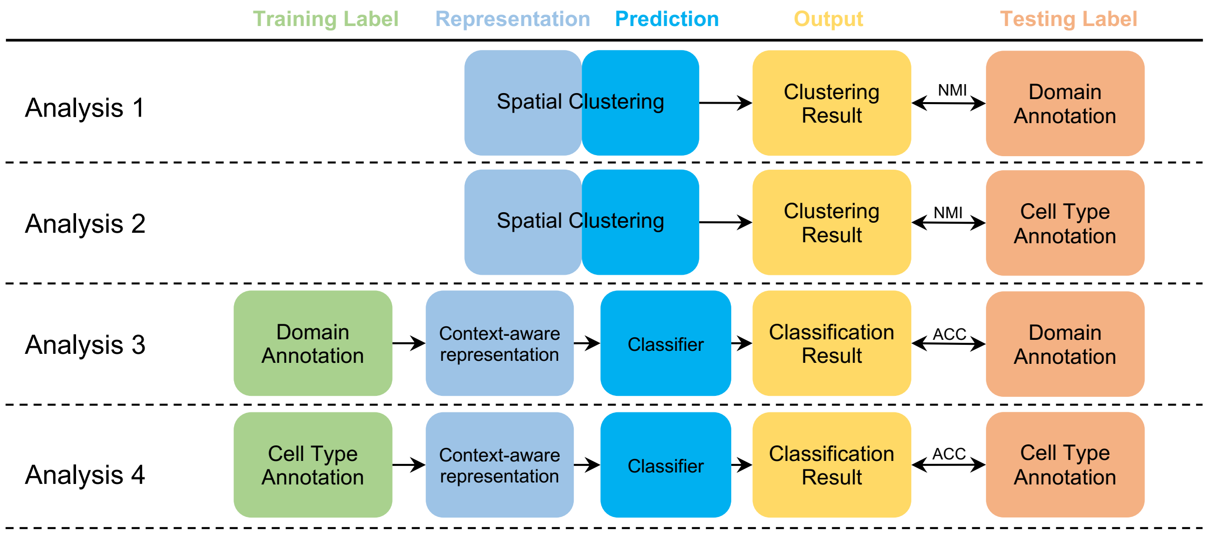

Supplementary Figure 15. 4 different analyses.
We performed 4 different analyses on MENDER performance. These analyses stem from different training/testing labels and the availability of supervision signal.
Analysis 1: MENDER is performed for unsupervised spatial domain identification, the performance is quantified using NMI between identified domains and domain annotation.
Analysis 2: MENDER is performed for unsupervised spatial domain identification, the performance is quantified using NMI between identified domains and cell type annotation.
Analysis 3: MENDER is performed for supervised spatial domain prediction, the performance is quantified using the prediction accuracy between predicted domain labels and domain annotation.
Analysis 4: MENDER is performed for supervised cell type prediction, the performance is quantified using the prediction accuracy between predicted cell types and cell type annotation.

Supplementary Figure 16

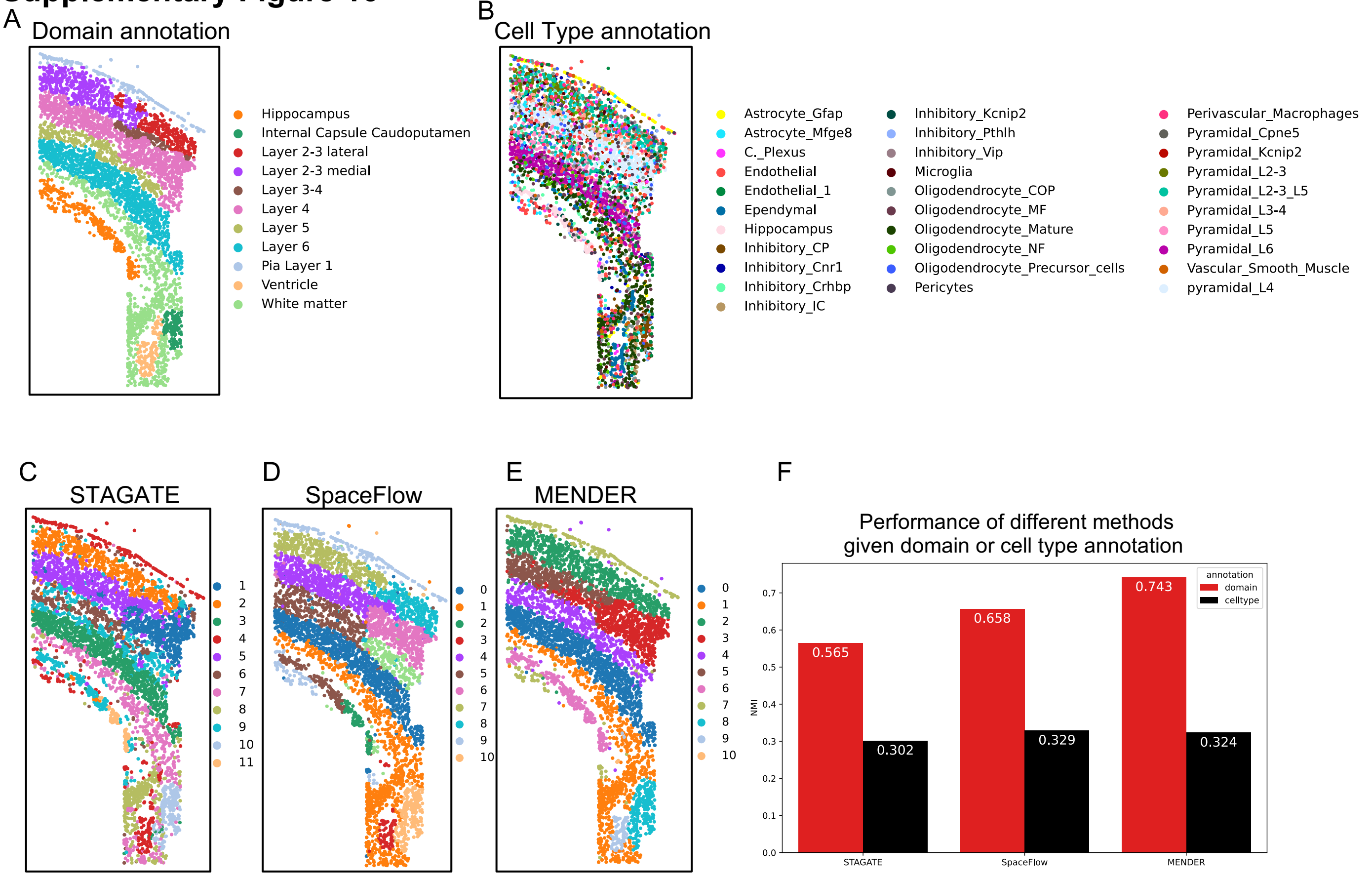

Supplementary Figure 16. Analysis 2 results of Supplementary Figure 16.

Methods comparison when evaluating using cell type annotation (osmFISH Somatosensory cortex data).

A: Domain annotation provided in (Codeluppi et al., Nature Methods, 2018).

B: Cell type annotation provided in (Codeluppi et al., Nature Methods, 2018).

C-E: Spatial clustering results of different methods, i.e., STAGATE (C), SpaceFlow (D), and MENDER (E).

F: Quantitative comparison of different methods. Red bar: the NMI is computed by using Domain annotation in (A) as ground truth; Black bar: the NMI is computed by using Cell Type annotation as ground truth.

Supplementary Figure 17

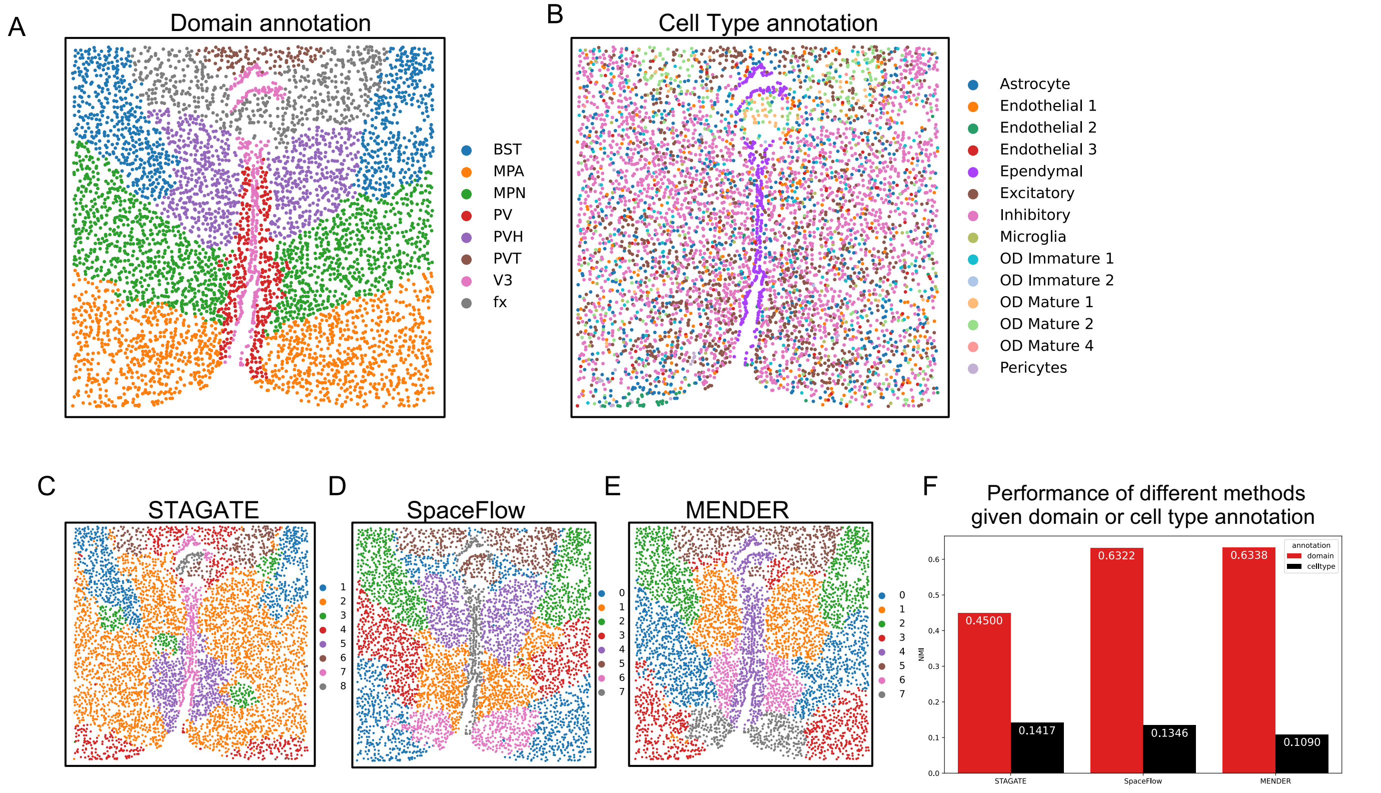

Supplementary Figure 17. Analysis 2 results of Supplementary Figure 15.
Methods comparison when evaluating using cell type annotation (MERFISH hypothalamic preoptic region data).
A: Domain annotation provided in (Li et al., Genome Biology, 2022).
B: Cell type annotation (Moffitt et al., Science, 2022).
C-E: Spatial clustering results of different methods, i.e., STAGATE (C), SpaceFlow (D), and MENDER (E).
F: Quantitative comparison of different methods. Red bar: the NMI is computed by using Domain annotation in (A) as ground truth; Black bar: the NMI is computed by using Cell Type annotation as ground truth.

Supplementary Figure 18

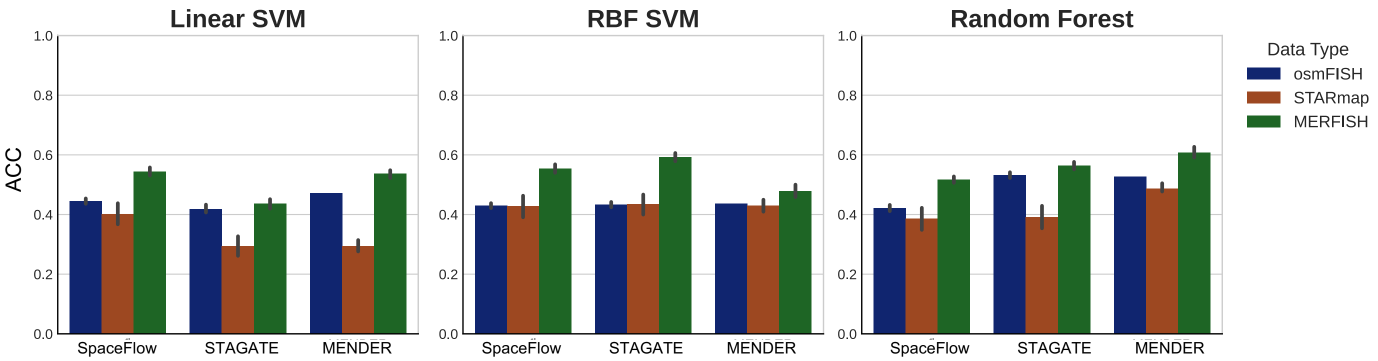

Supplementary Figure 18. Analysis 4 results of Supplementary Figure 15.
We applied supervised classifiers on the context-aware representation generated by different methods, i.e., SpaceFlow, STAGATE, and MENDER, using the cell type annotation as the supervision signal for each cell. The classification accuracy (sklearn.metrics.accuracy_score implementation) was reported as the median value from 5-fold cross-validation. Three different data types were used, including osmFISH, STARmap, and MERFISH. Source data are provided as a Source Data file Source data are provided as a Source Data file.

Supplementary Figure 19

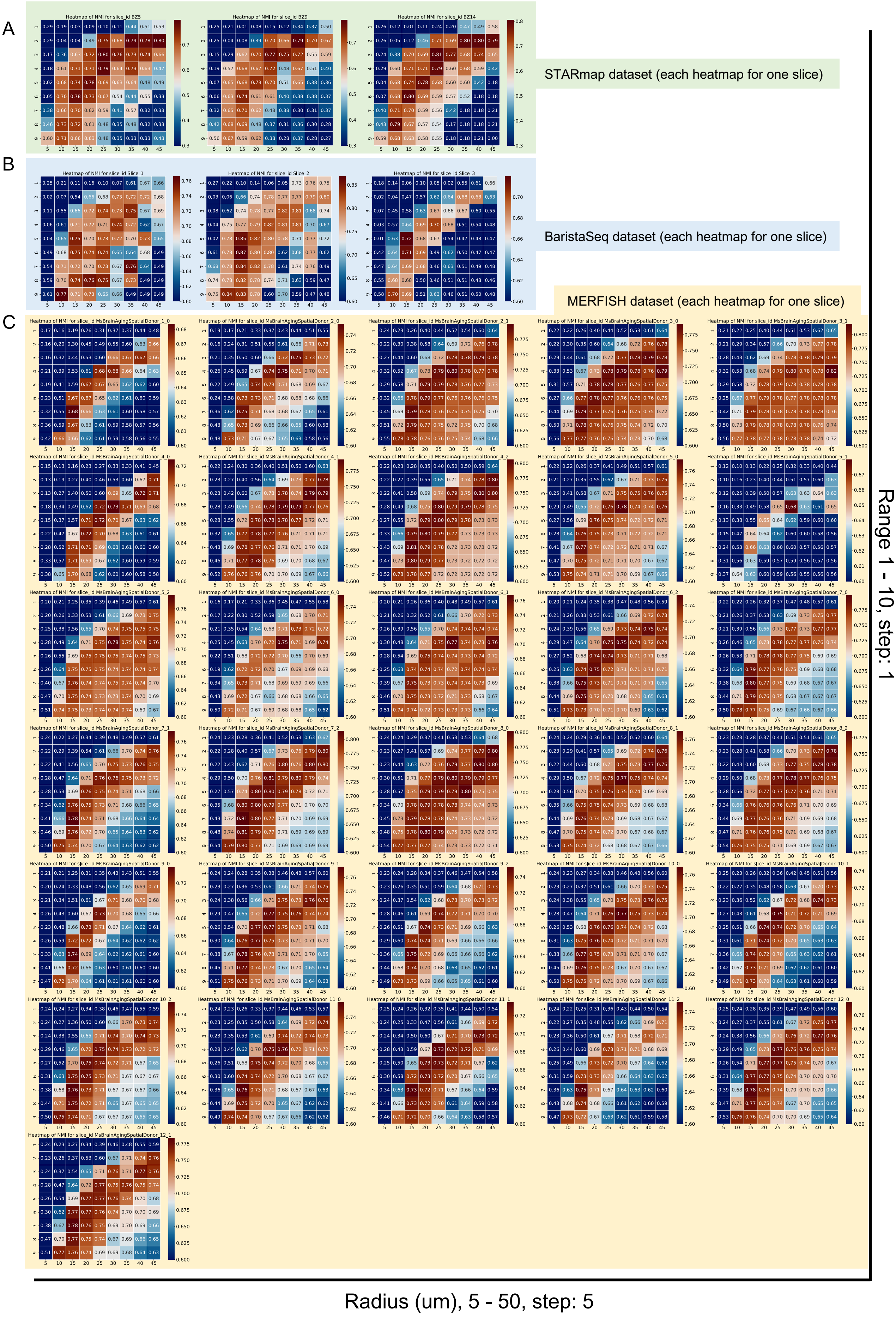

Supplementary Figure 19. Evaluation of MENDER Performance Under Various Parameter Settings. This figure presents the influence of varying parameters on the performance of MENDER, quantified by Normalized Mutual Information (NMI), across 3 benchmark datasets: STARmap (A), BaristaSeq (B), and MERFISH (C). Specifically, for the STARmap dataset (A) exemplified here, the three displayed heatmaps correspond to three different slices of the data. Each heatmap illustrates the influence of the Radius (horizontal axis) and Range (vertical axis) parameters on the performance of MENDER. The same is true for (B) and (C). Source data are provided as a Source Data file Source data are provided as a Source Data file.

Supplementary Figure 20

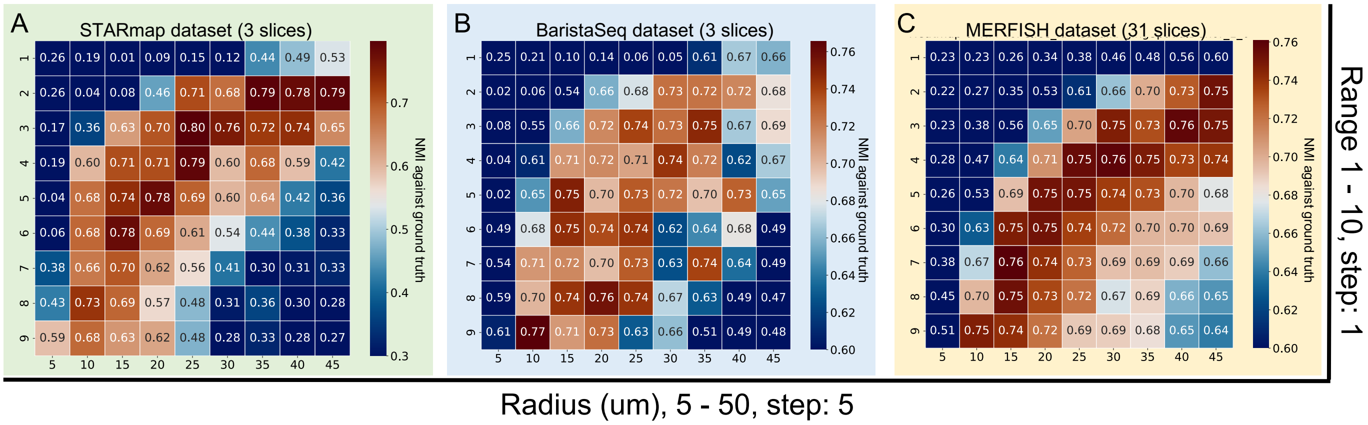

Supplementary Figure 20. Evaluation of MENDER Performance Under Various Parameter Settings.
This figure presents the influence of varying parameters on the performance of MENDER, quantified by Normalized Mutual Information (NMI), across three benchmark datasets: STARmap (A), BaristaSeq (B), and MERFISH (C). Specifically, for the STARmap dataset (A) exemplified here, the displayed heatmap summarizes the median NMI of three slices. The heatmap illustrates the influence of the Radius (horizontal axis) and Range (vertical axis) parameters on the performance of MENDER. The similar is true for (B) and (C).

Supplementary Figure 21

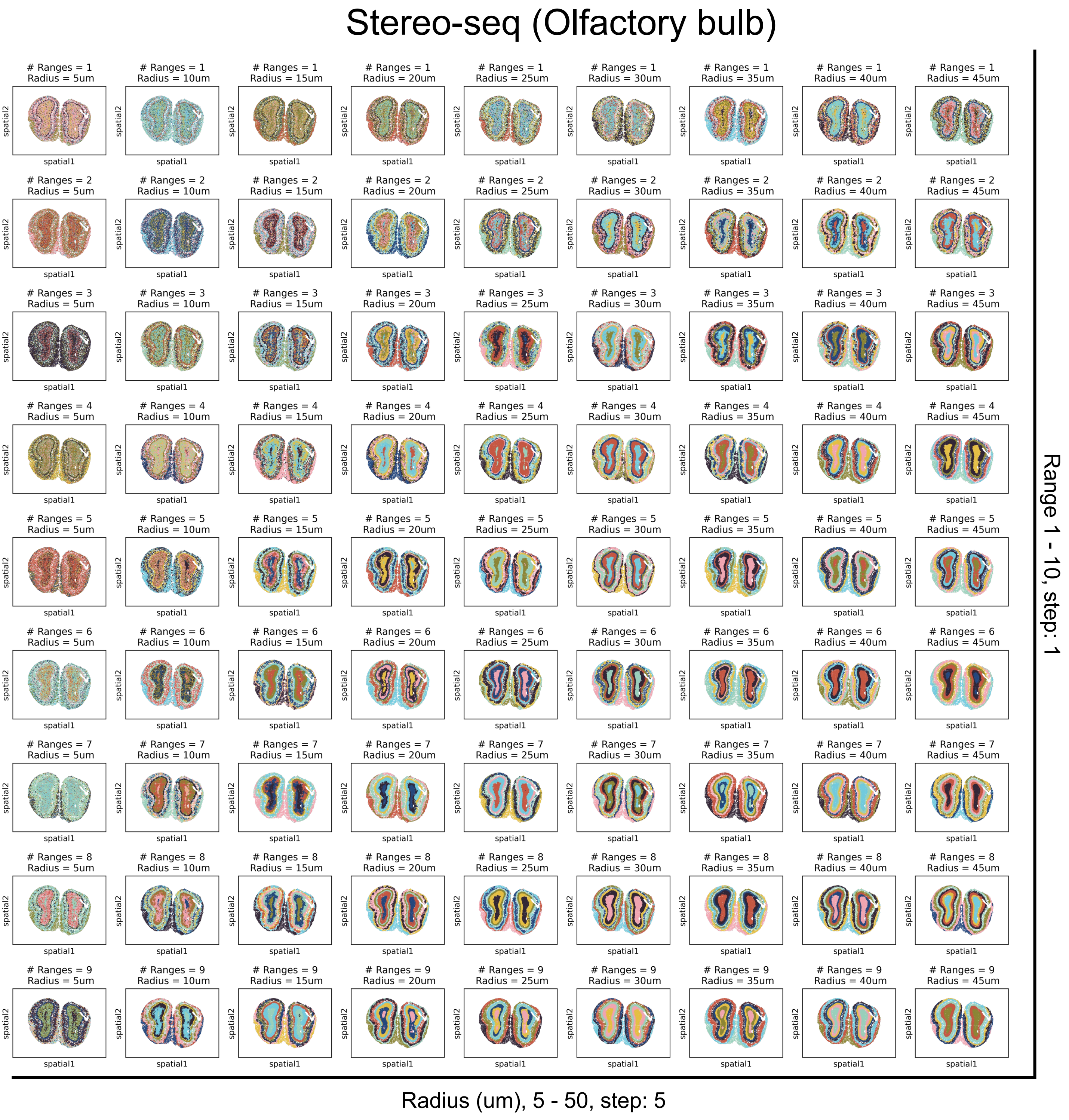

Supplementary Figure 21. Evaluation of MENDER Performance Under Various Parameter Settings (Stereo-seq). This figure uses Stereo-seq data to show the influence of varying parameters on the performance of MENDER. Rows represent different Range values and columns represent different Radius values. Source data are provided as a Source Data file Source data are provided as a Source Data file.

Supplementary Figure 22

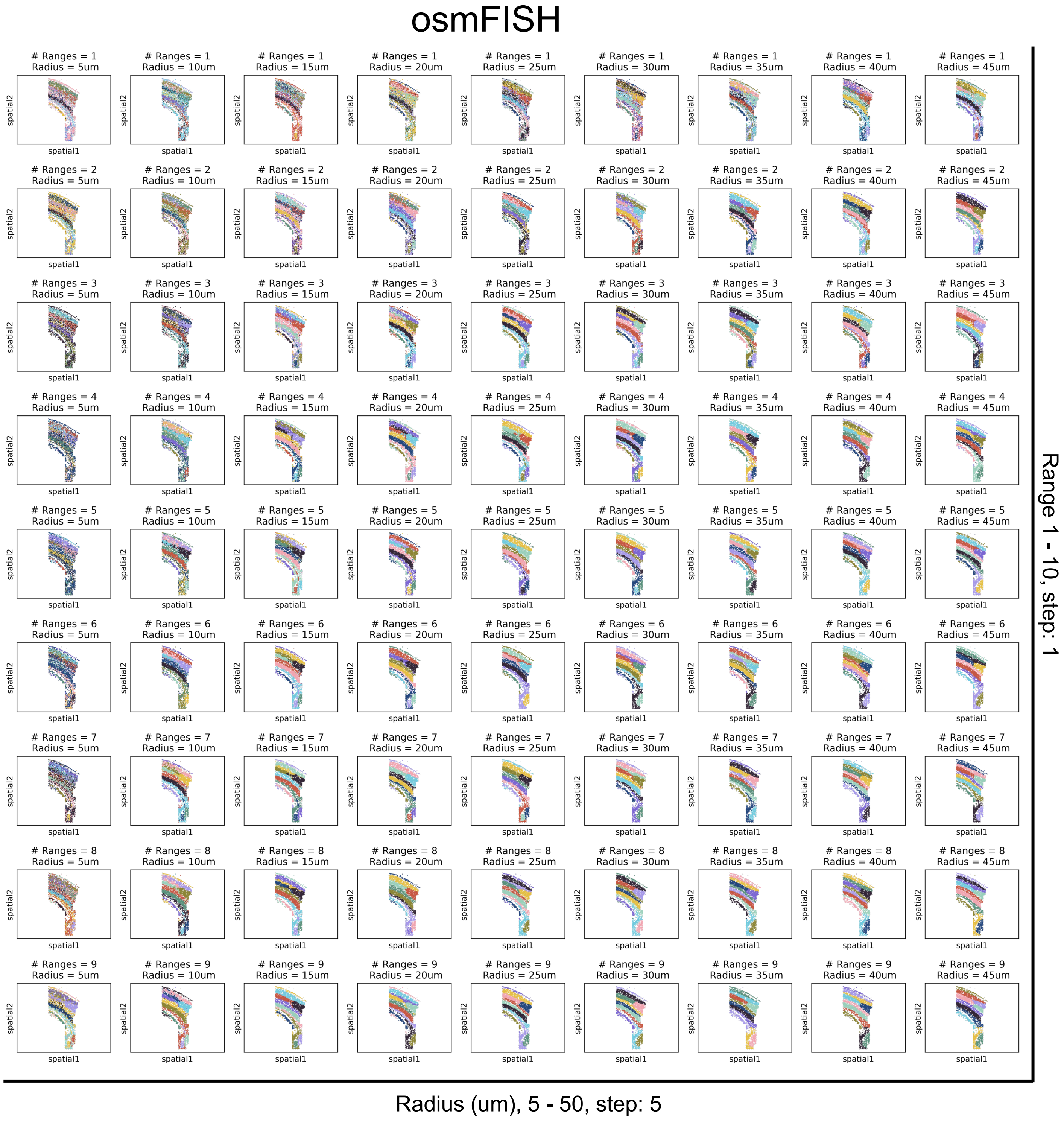

Supplementary Figure 22. Evaluation of MENDER Performance Under Various Parameter Settings (osmFISH).
This figure uses osmFISH data to show the influence of varying parameters on the performance of MENDER. Rows represent different Range values and columns represent different Radius values. Source data are provided as a Source Data file Source data are provided as a Source Data file.

Supplementary Figure 23

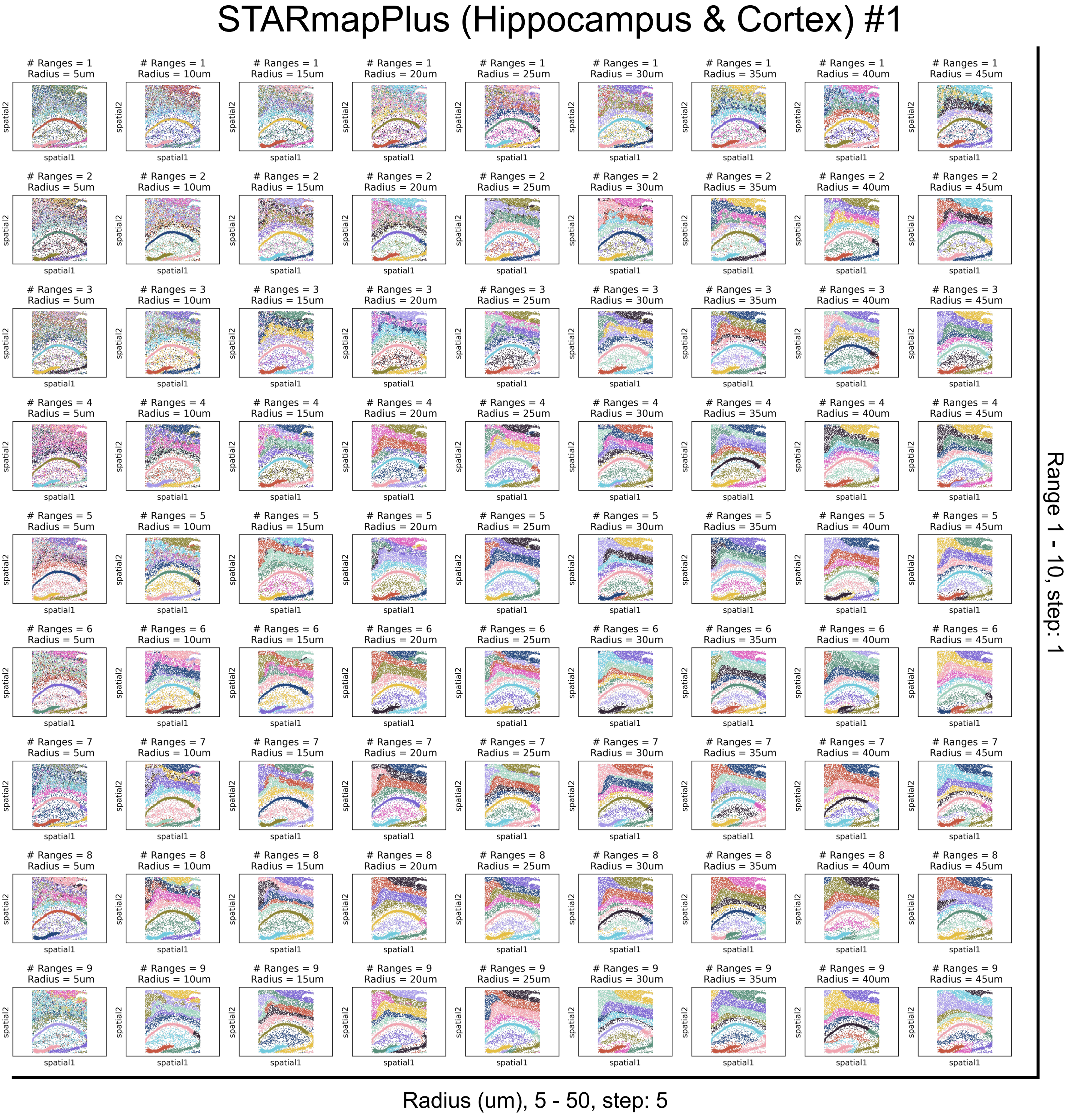

Supplementary Figure 23. Evaluation of MENDER Performance Under Various Parameter Settings (STARmapPLUS sample 1). This figure uses STARmapPlus data to show the influence of varying parameters on the performance of MENDER. Rows represent different Range values and columns represent different Radius values.

Supplementary Figure 24

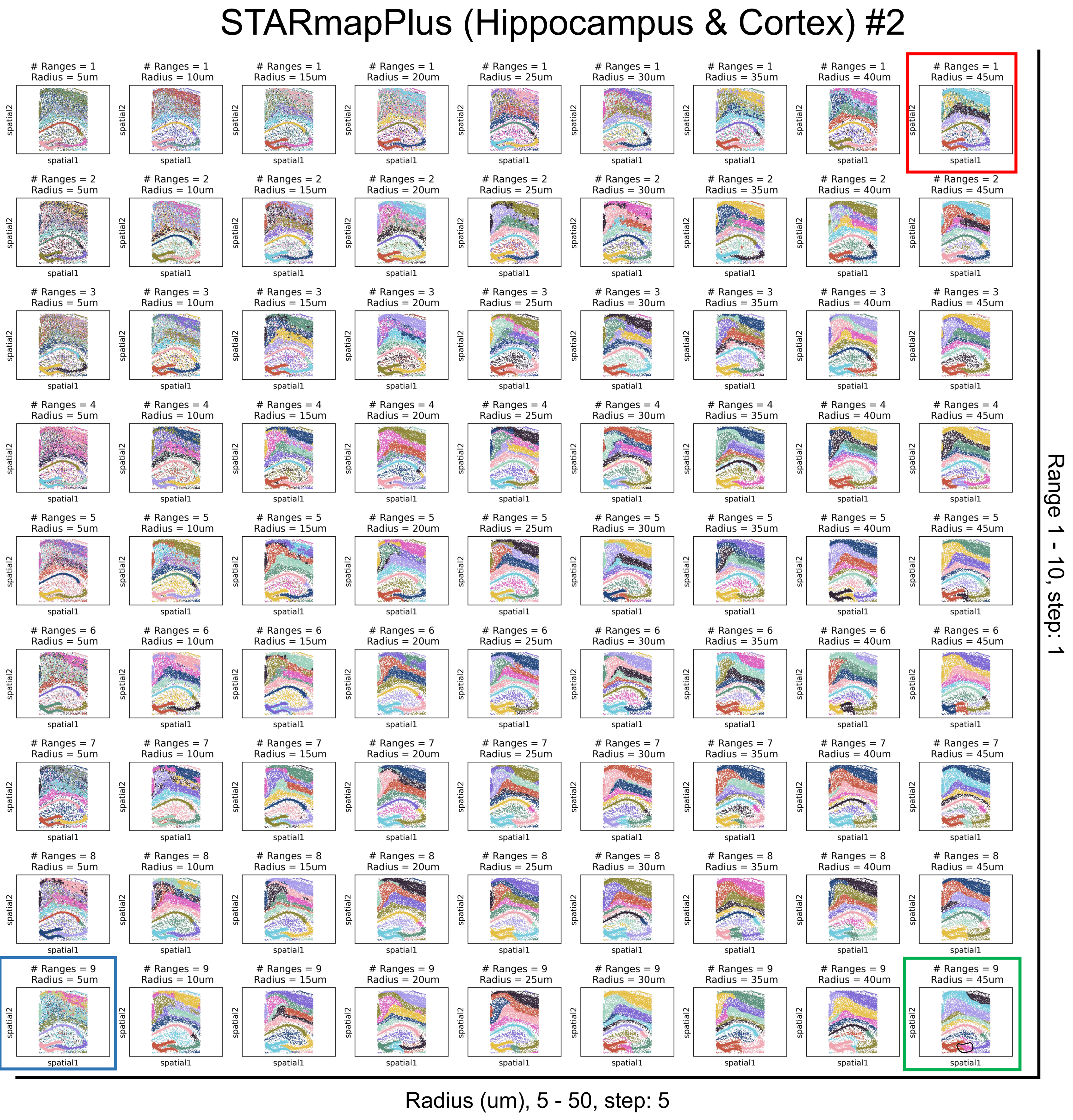

Supplementary Figure 24. Evaluation of MENDER Performance Under Various Parameter Settings (STARmapPLUS sample 2). This figure uses STARmapPlus data to show the influence of varying parameters on the performance of MENDER. Rows represent different Range values and columns represent different Radius values.

Supplementary Figure 25

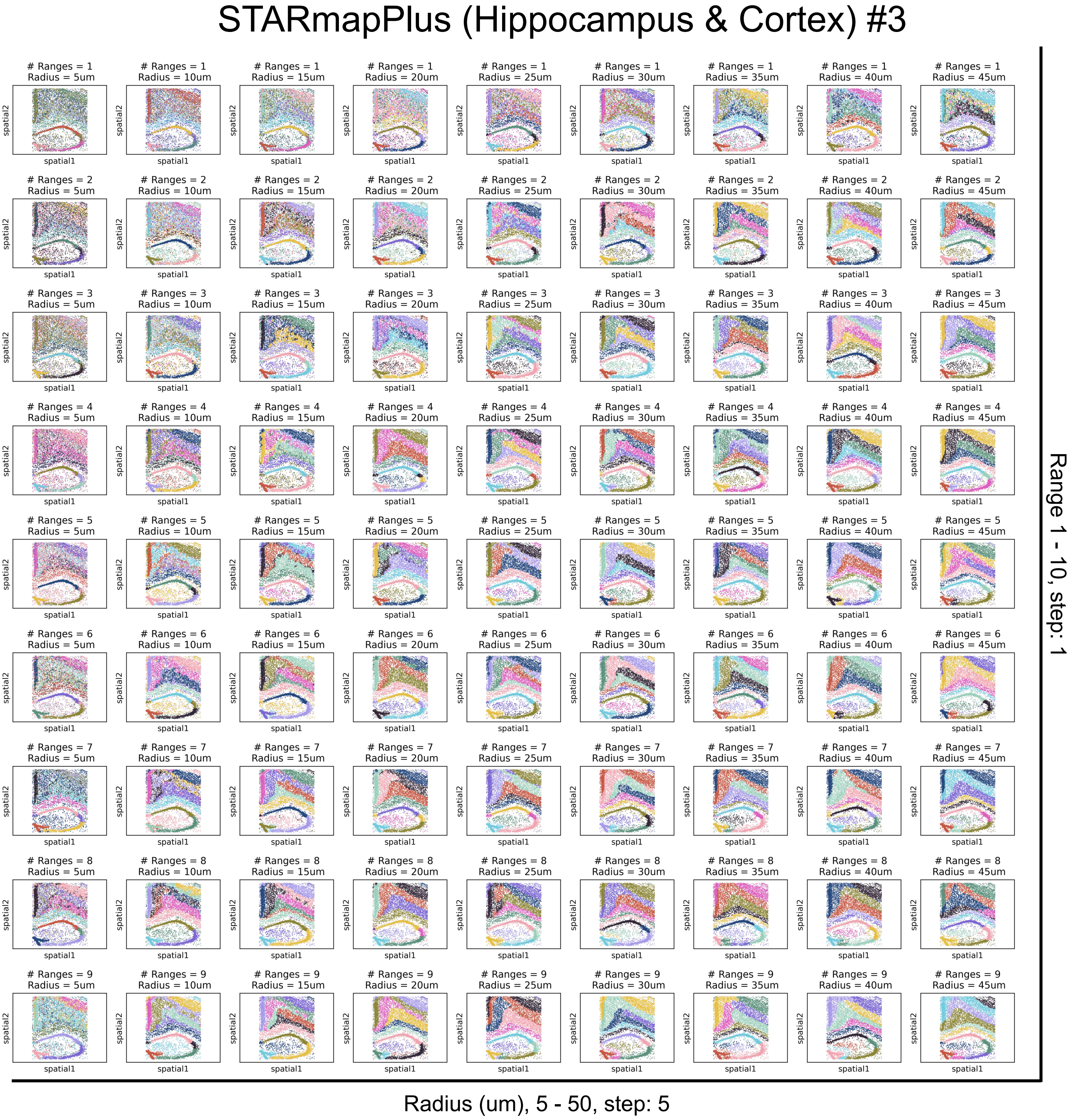

Supplementary Figure 25. Evaluation of MENDER Performance Under Various Parameter Settings (STARmapPLUS sample 3). This figure uses STARmapPlus data to show the influence of varying parameters on the performance of MENDER. Rows represent different Range values and columns represent different Radius values.

Supplementary Figure 26

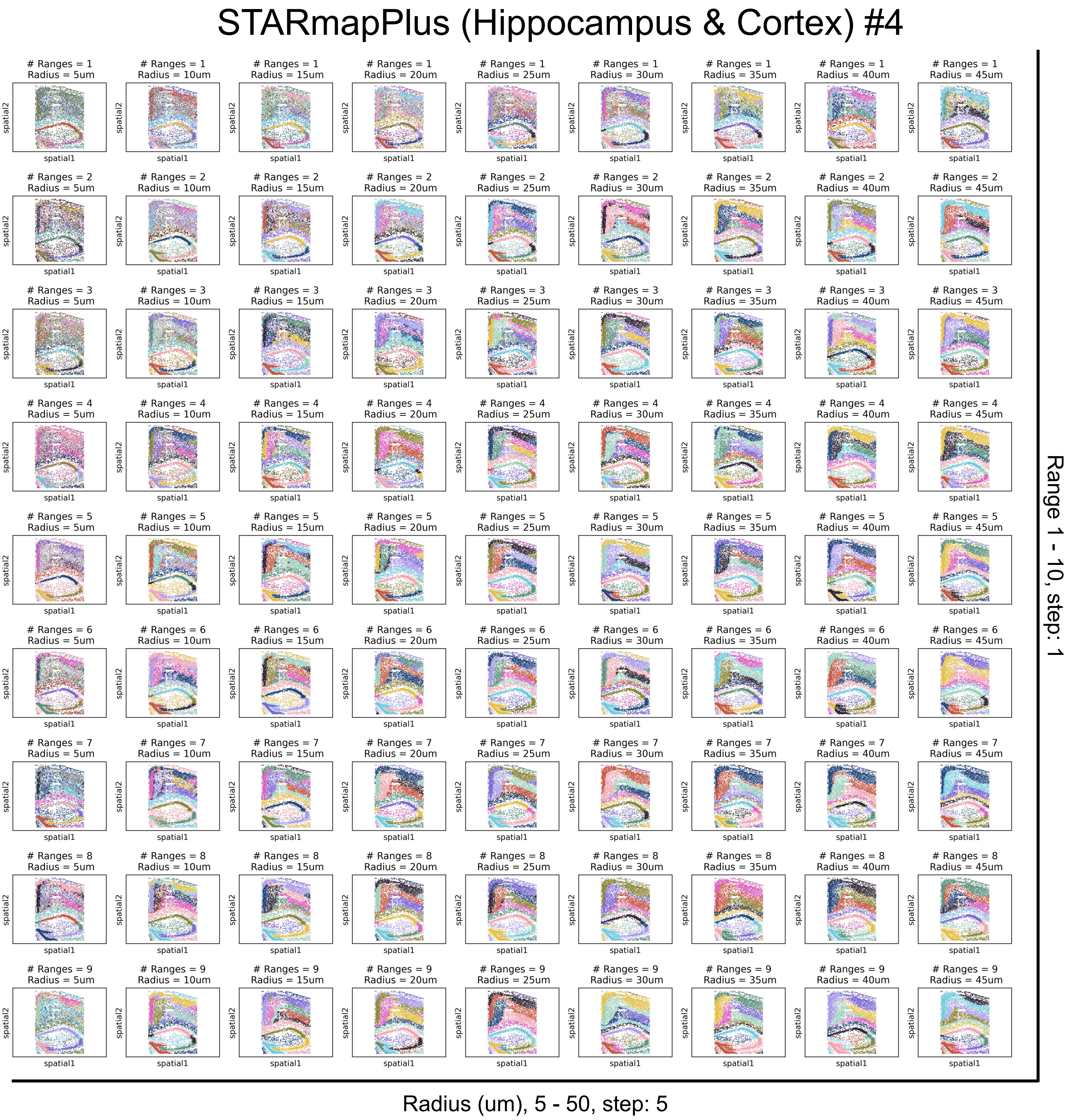

Supplementary Figure 26. Evaluation of MENDER Performance Under Various Parameter Settings (STARmapPLUS sample 4). This figure uses STARmapPlus data to show the influence of varying parameters on the performance of MENDER. Rows represent different Range values and columns represent different Radius values.

Supplementary Figure 27

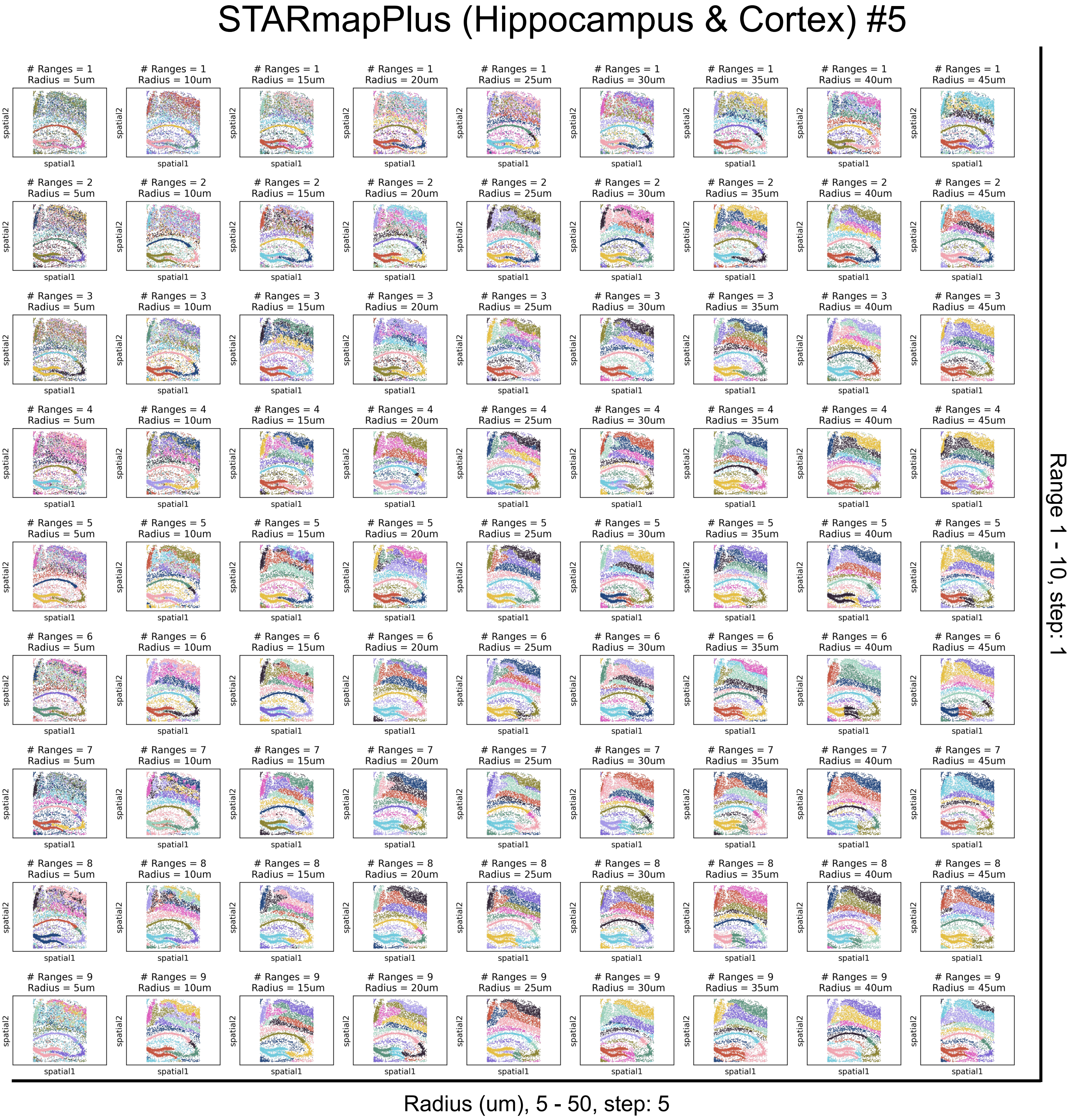

Supplementary Figure 27. Evaluation of MENDER Performance Under Various Parameter Settings (STARmapPLUS sample 5).
This figure uses STARmapPlus data to show the influence of varying parameters on the performance of MENDER. Rows represent different Range values and columns represent different Radius values.

Supplementary Figure 28

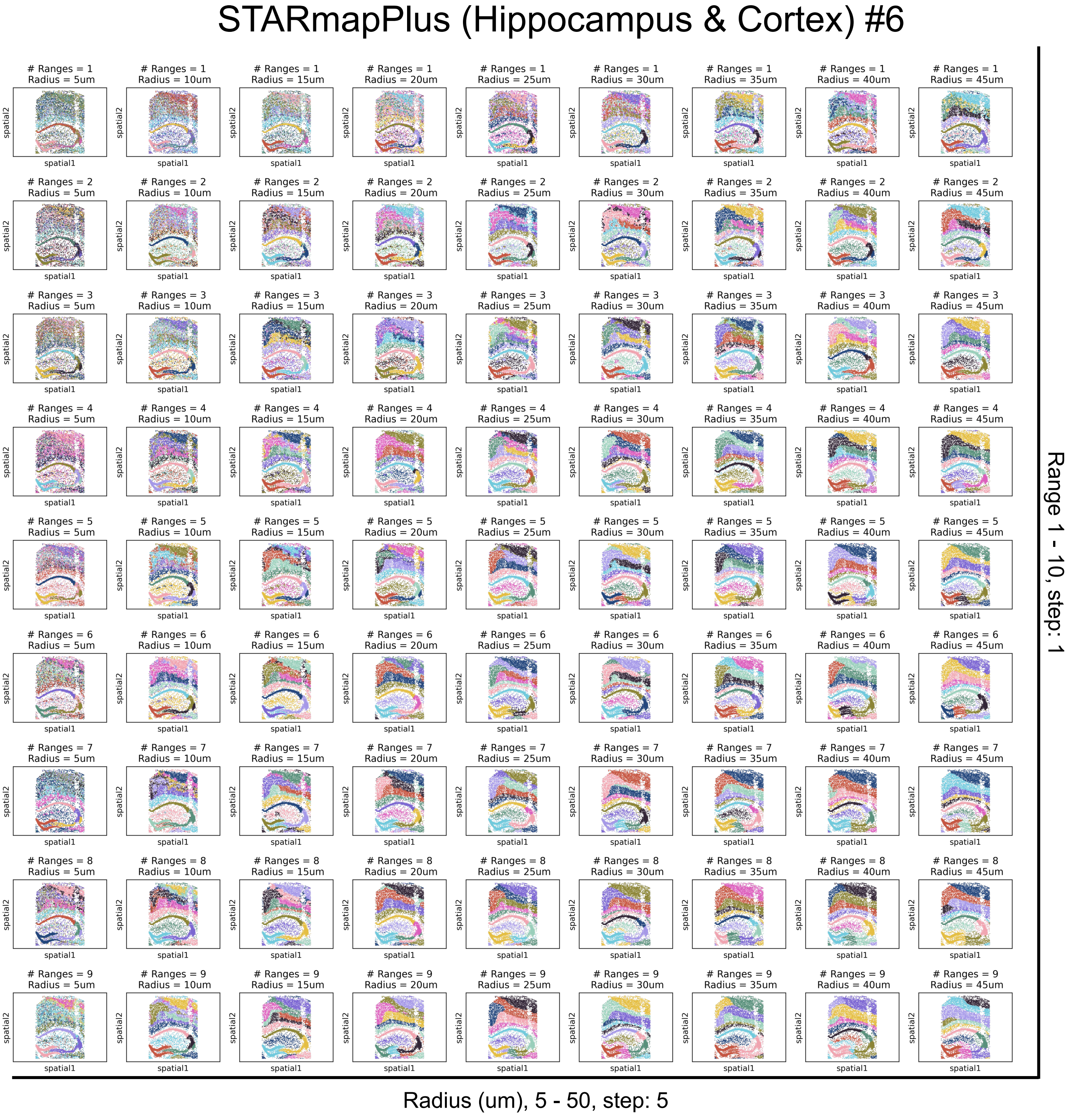

Supplementary Figure 28. Evaluation of MENDER Performance Under Various Parameter Settings (STARmapPLUS sample 6).
This figure uses STARmapPlus data to show the influence of varying parameters on the performance of MENDER. Rows represent different Range values and columns represent different Radius values.

Supplementary Figure 29

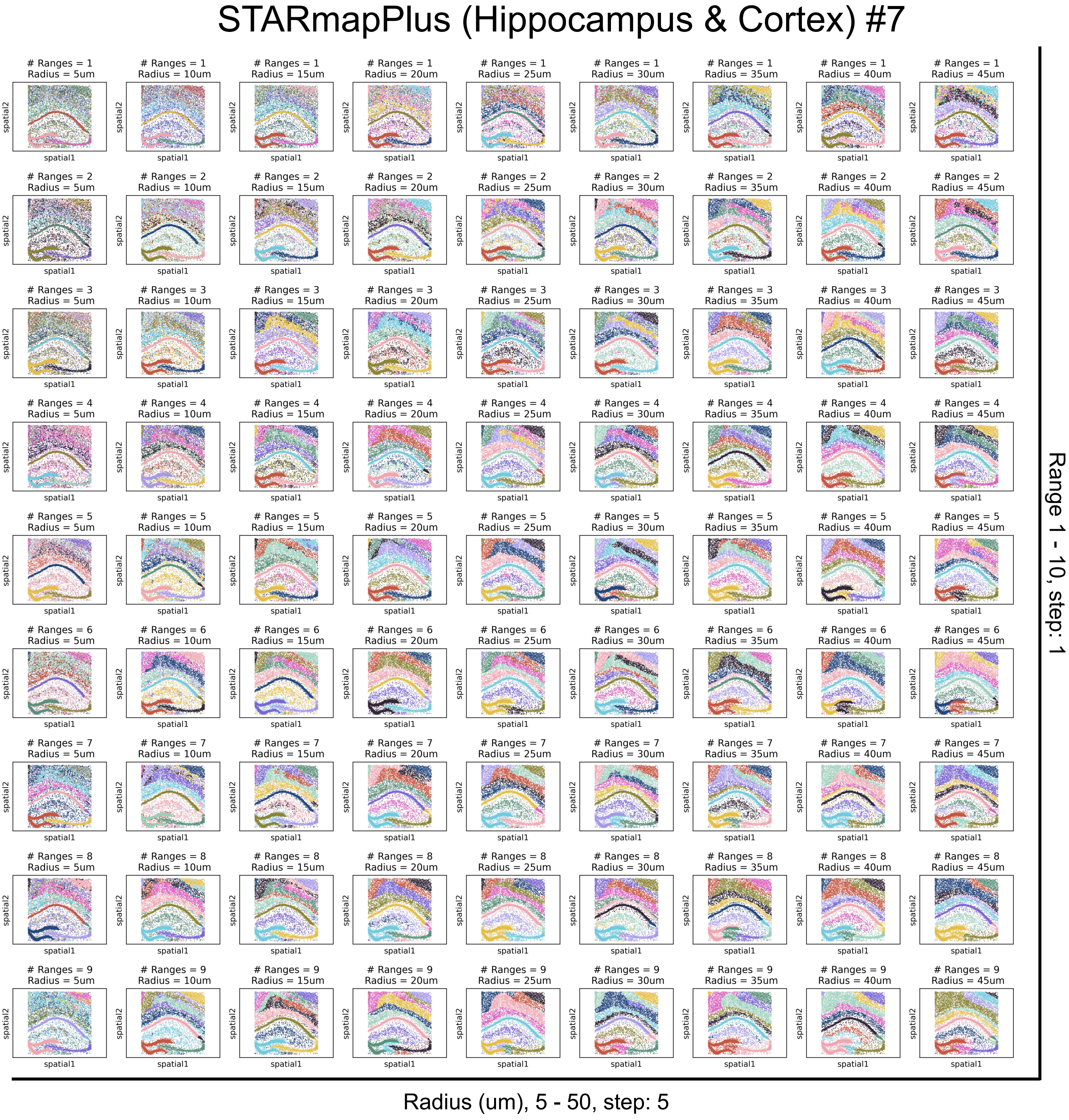

Supplementary Figure 29. Evaluation of MENDER Performance Under Various Parameter Settings (STARmapPLUS sample 7). This figure uses STARmapPlus data to show the influence of varying parameters on the performance of MENDER. Rows represent different Range values and columns represent different Radius values.

Supplementary Figure 30

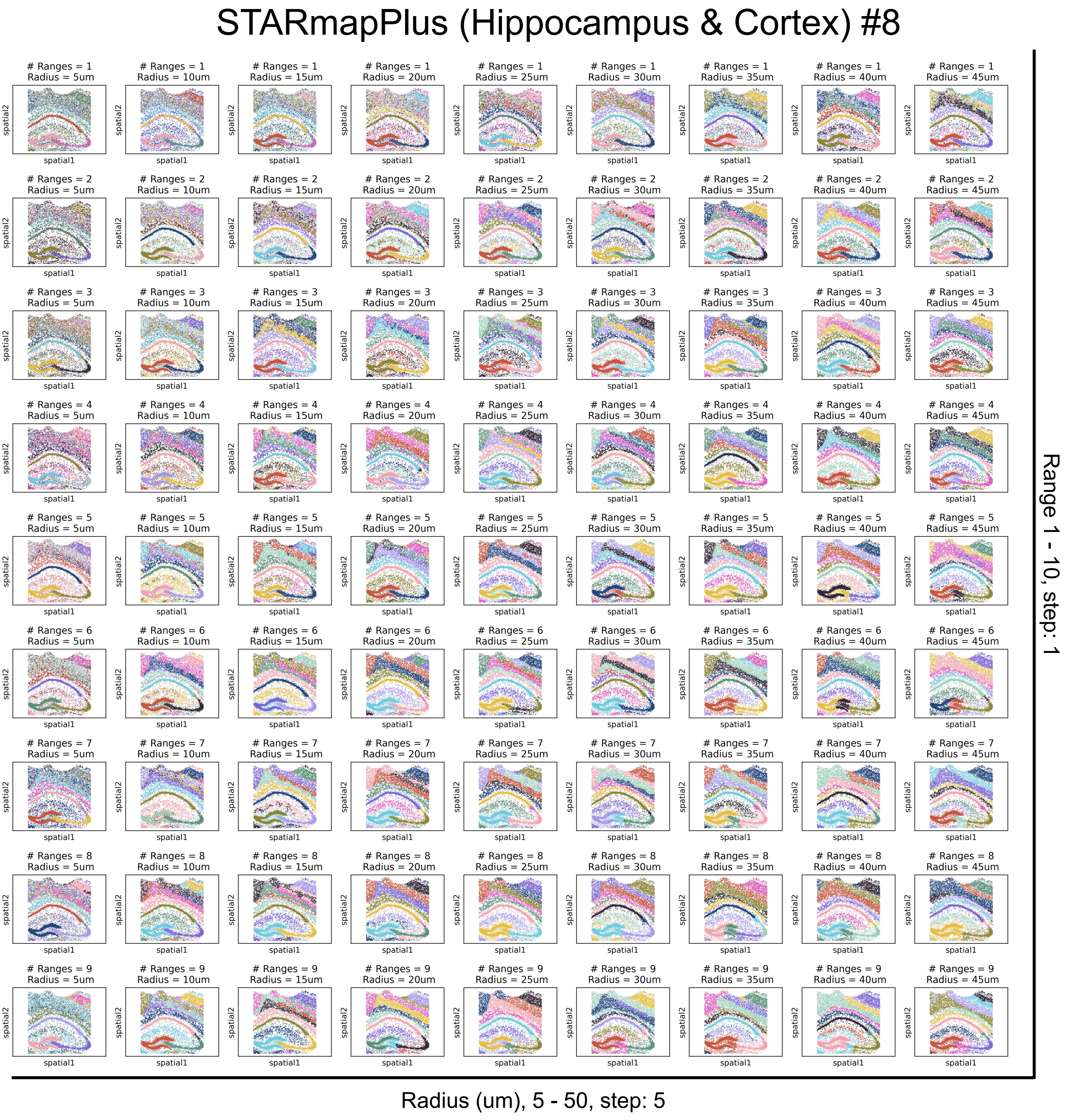

Supplementary Figure 30. Evaluation of MENDER Performance Under Various Parameter Settings (STARmapPLUS sample 8). This figure uses STARmapPlus data to show the influence of varying parameters on the performance of MENDER. Rows represent different Range values and columns represent different Radius values.

Supplementary Figure 31

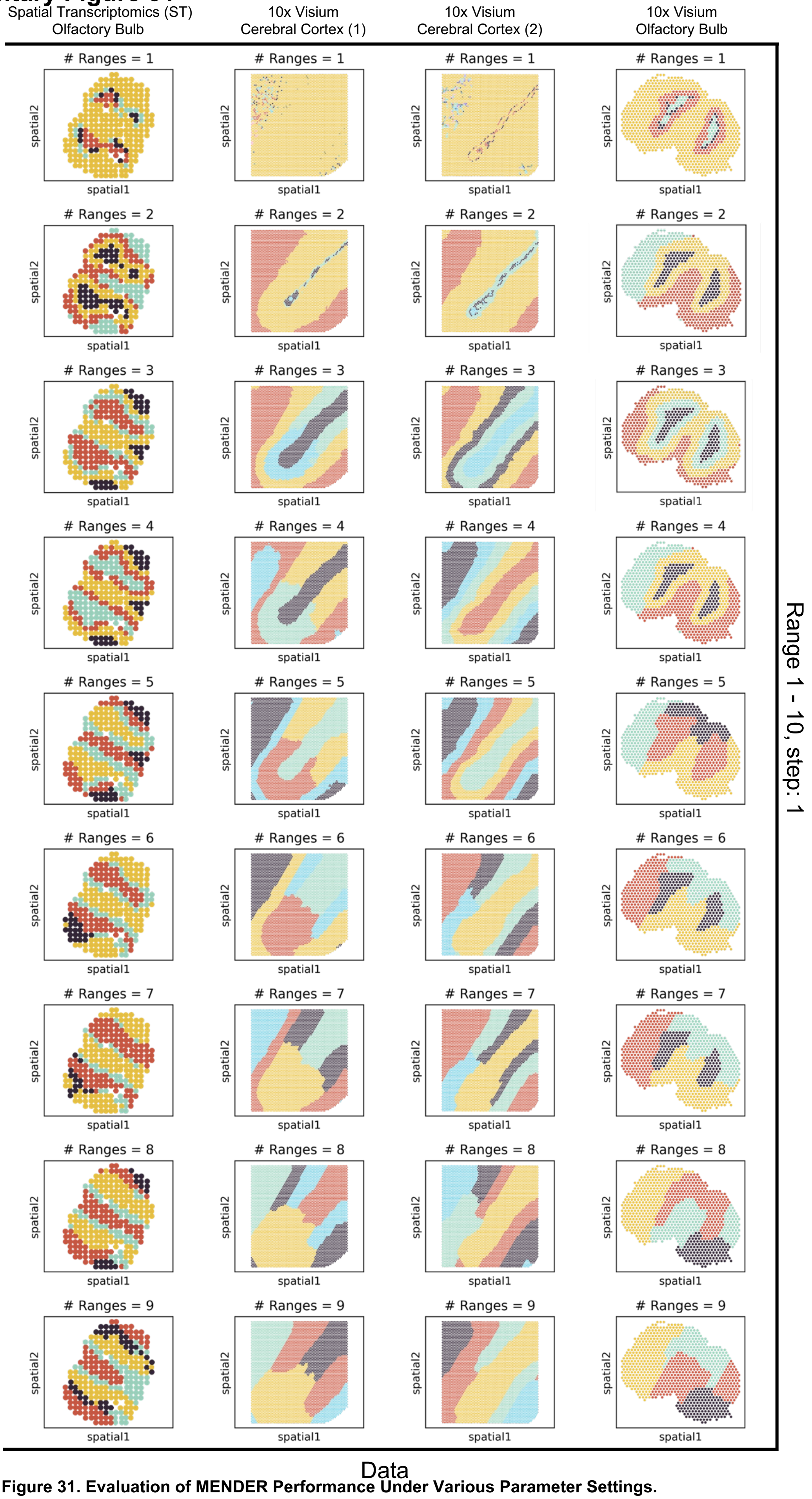

Supplementary Figure 31. Evaluation of MENDER Performance Under Various Parameter Settings.
This figure illustrates the influence of varying parameters on the performance of MENDER using different datasets. The datasets include the Spatial Transcriptomics Olfactory Bulb data (first column), 10x Visium Cerebral Cortex data (second and third columns, representing two replicate data), and 10x Visium Olfactory Bulb data (fourth column). For these datasets (grid-like spatial distribution of spots), the Radius parameter does not need to be set. We examined a range of values from 1 to 10 for the Range parameter, with each row representing a different Range parameter. Source data are provided as a Source Data file Source data are provided as a Source Data file.

Supplementary Figure 32

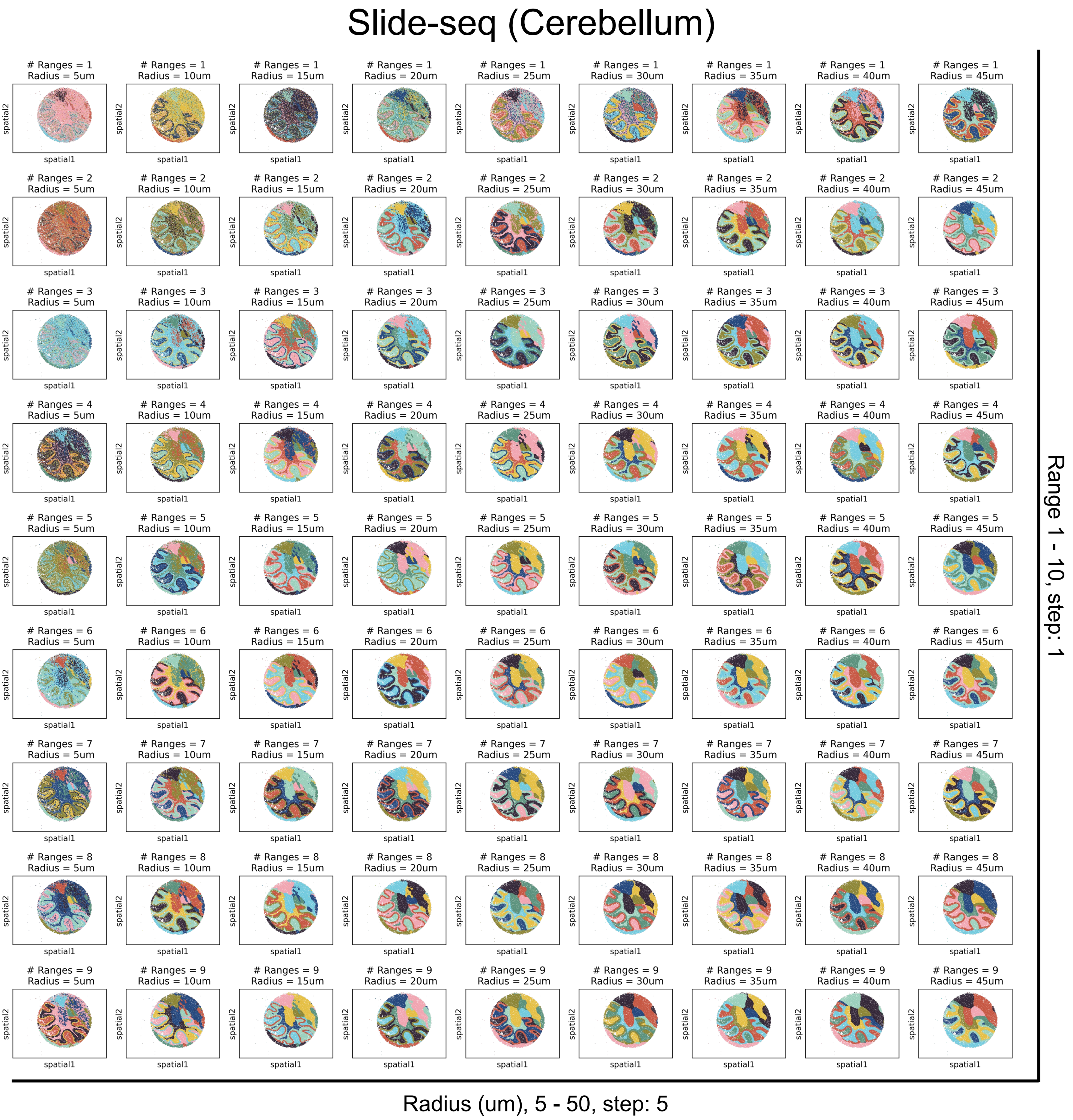

Supplementary Figure 32. Evaluation of MENDER Performance Under Various Parameter Settings (Slide-seq Cerebellum).
This figure uses Slide-seq data to show the influence of varying parameters on the performance of MENDER. Rows represent different Range values and columns represent different Radius values. Source data are provided as a Source Data file Source data are provided as a Source Data file.

Supplementary Figure 33

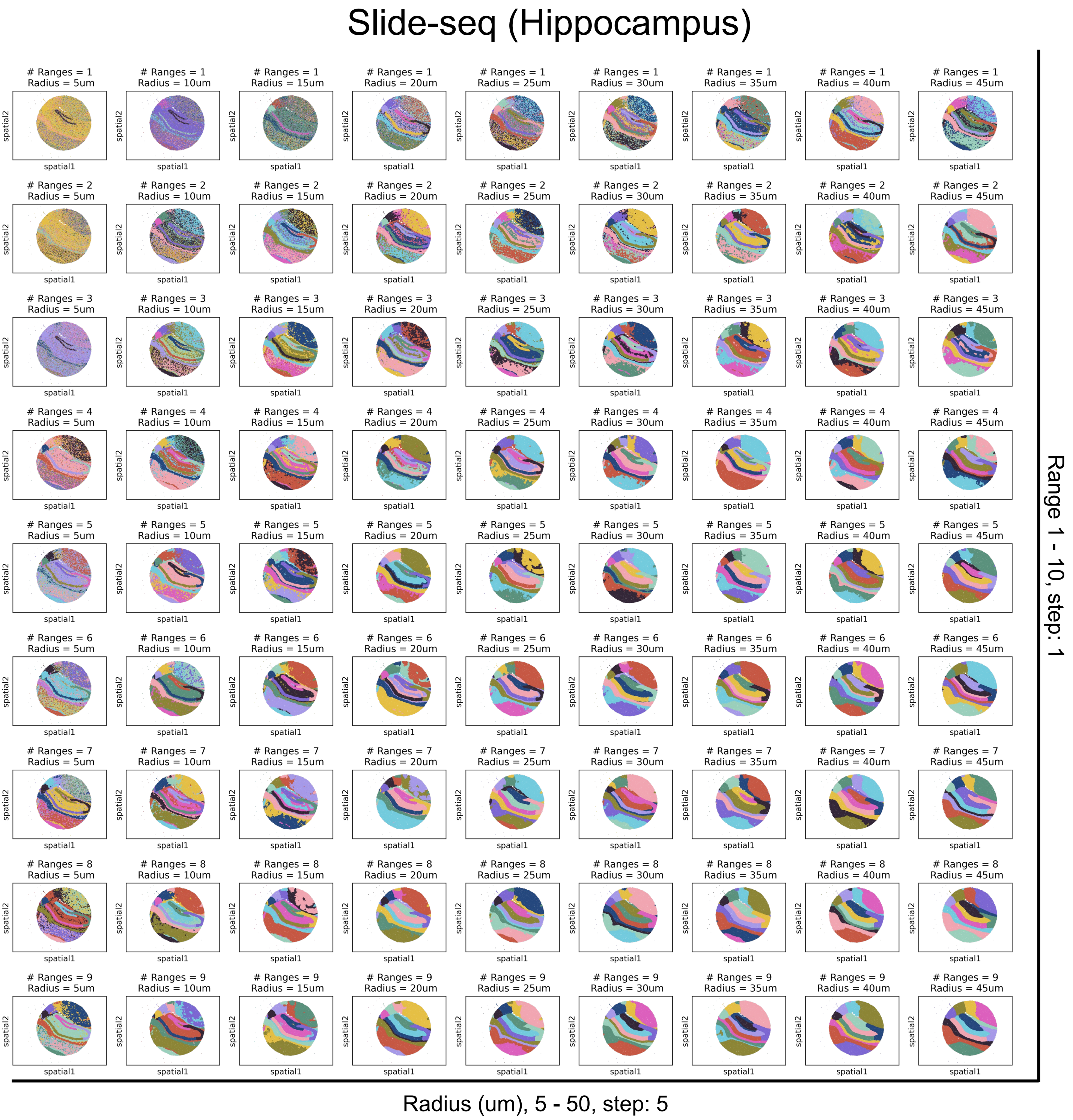

Supplementary Figure 33. Evaluation of MENDER Performance Under Various Parameter Settings (Slide-seq Hippocampus). This figure uses Slide-seq data to show the influence of varying parameters on the performance of MENDER. Rows represent different Range values and columns represent different Radius values. Source data are provided as a Source Data file Source data are provided as a Source Data file.

Supplementary Figure 34

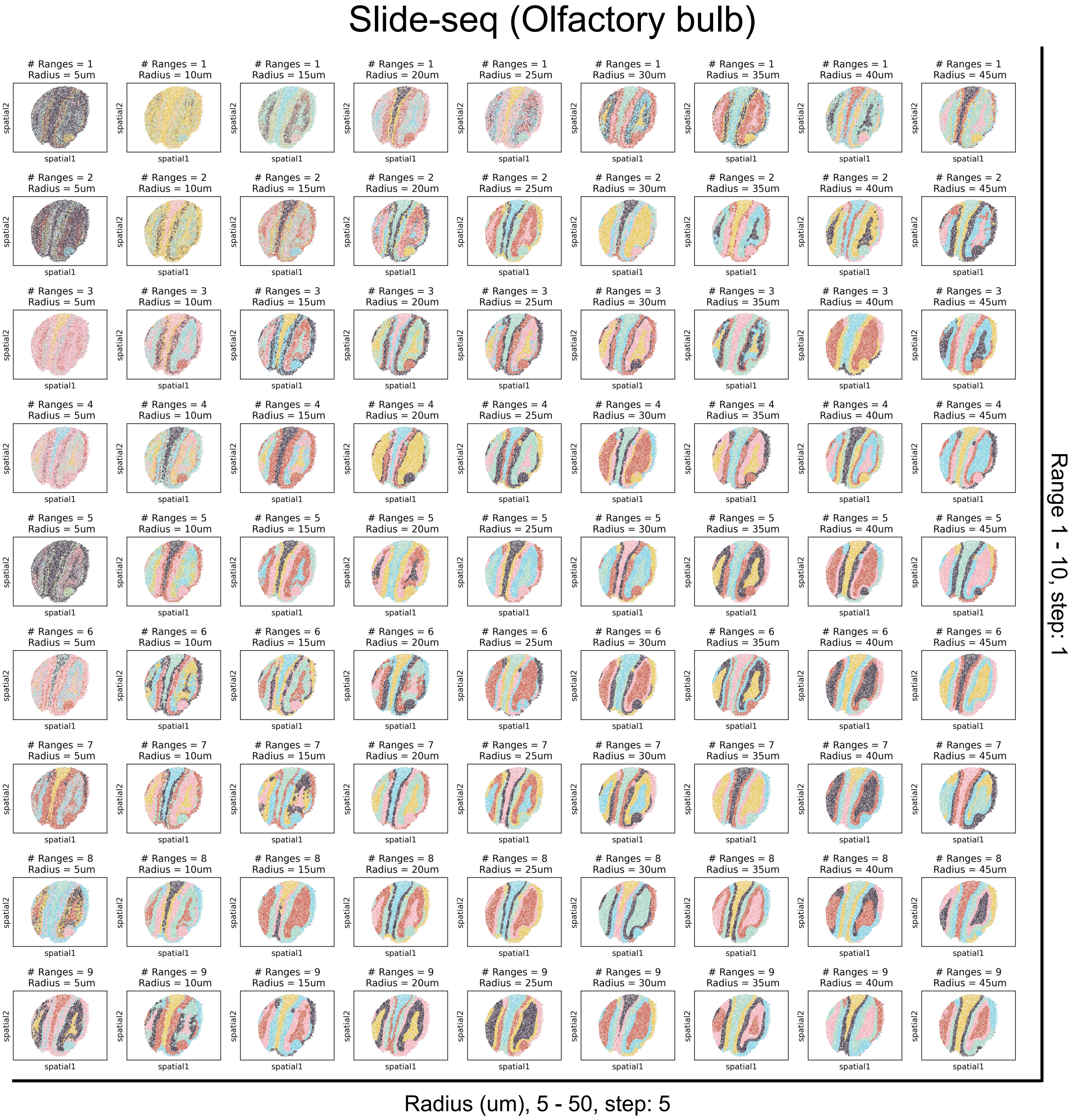

Supplementary Figure 34. Evaluation of MENDER Performance Under Various Parameter Settings (Slide-seq Olfactory bulb). This figure uses Slide-seq data to show the influence of varying parameters on the performance of MENDER. Rows represent different Range values and columns represent different Radius values. Source data are provided as a Source Data file Source data are provided as a Source Data file.

Supplementary Figure 35

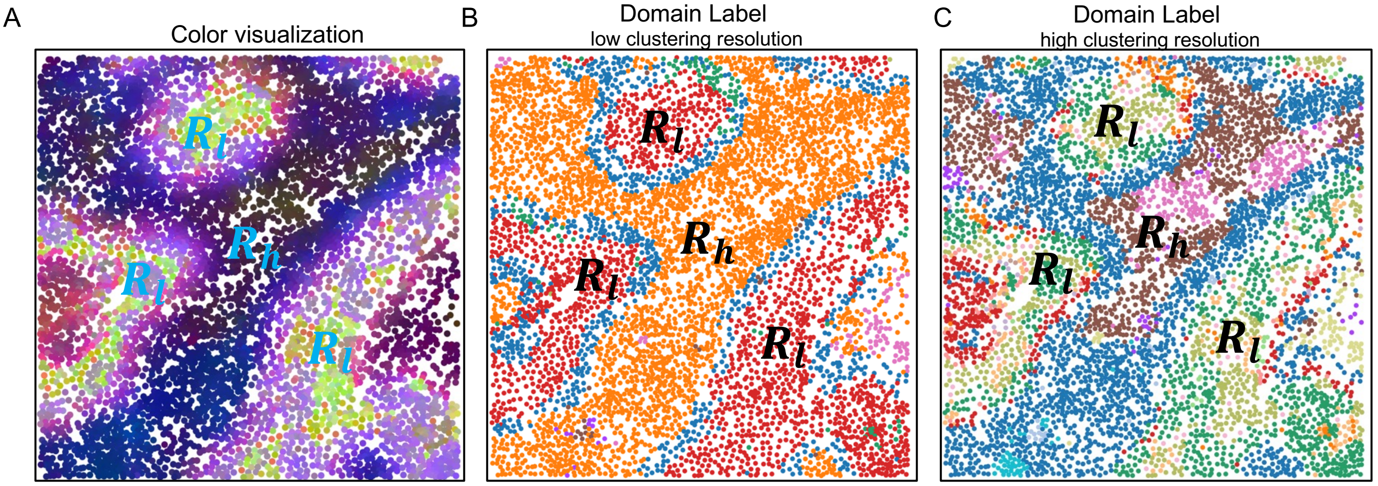

Supplementary Figure 35. Variations in Cellular context and spatial domains.
This same triple negative breast cancer data in Figure 6.
A: The color visualization is obtained by: (1) use UMAP dimensional reduction to reduce the high-dimensional cellular context representation obtained by MENDER to three dimensions. (2) assign each cell a color by linearly mapping its associated cellular context's 3D embedding to the CIELAB color space.
B: Domain labels obtained by decreased Leiden clustering resolution.
C: Domain labels obtained by increased Leiden clustering resolution

Supplementary Figure 36

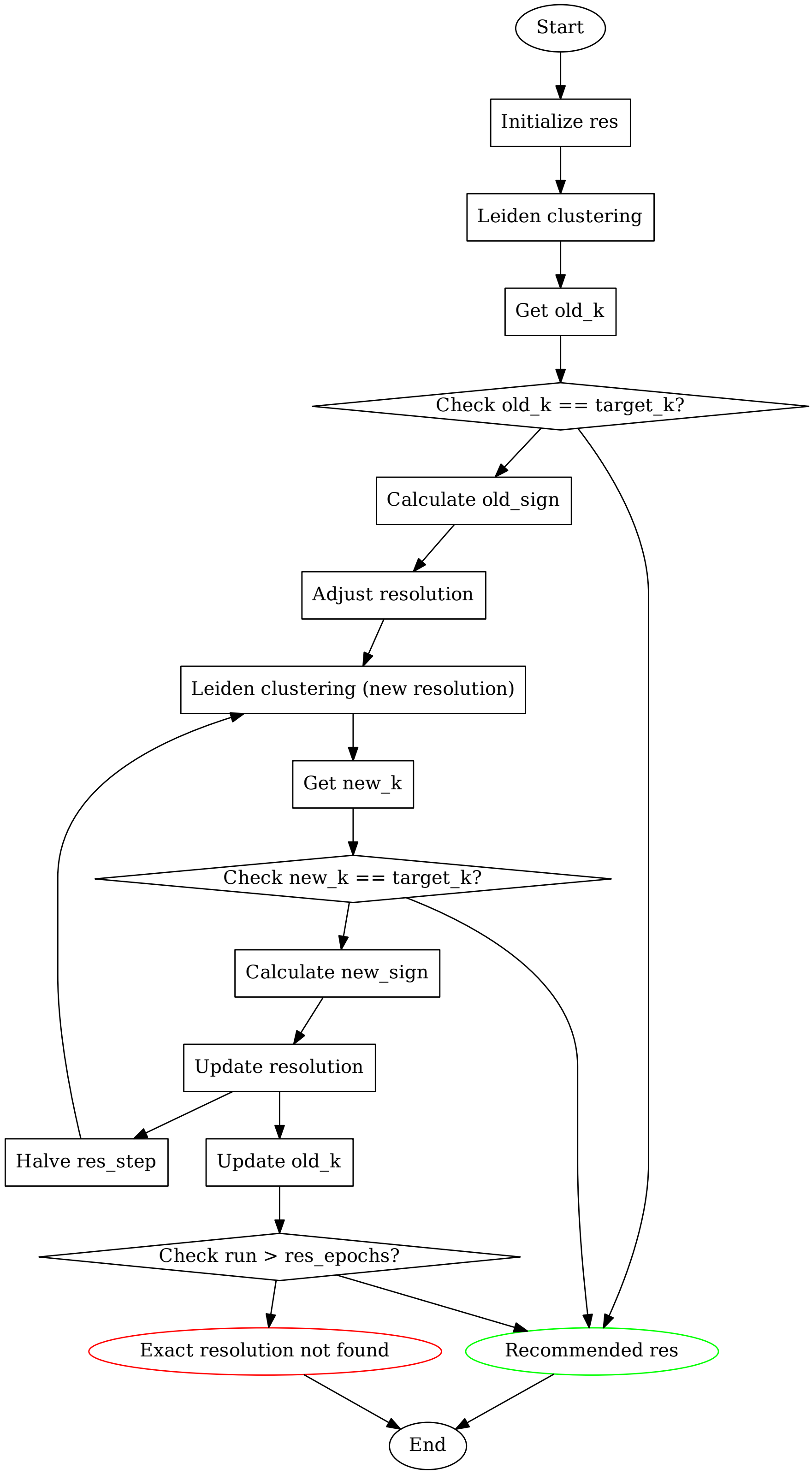

Supplementary Figure 36. Diagram of ”res_search” function.
When the clustering resolution is unknown, MENDER addressed the resolution issue by searching for the optimal Leiden resolution based on the expected number of regions. This function accepts several parameters, including "adata" (the dataset for clustering), "target_k" (the expected number of regions), "res_start" (initial clustering resolution), "res_step" (search step), "res_epochs" (maximum search epochs), and "random_state" (random seed).

Supplementary Figure 37

A

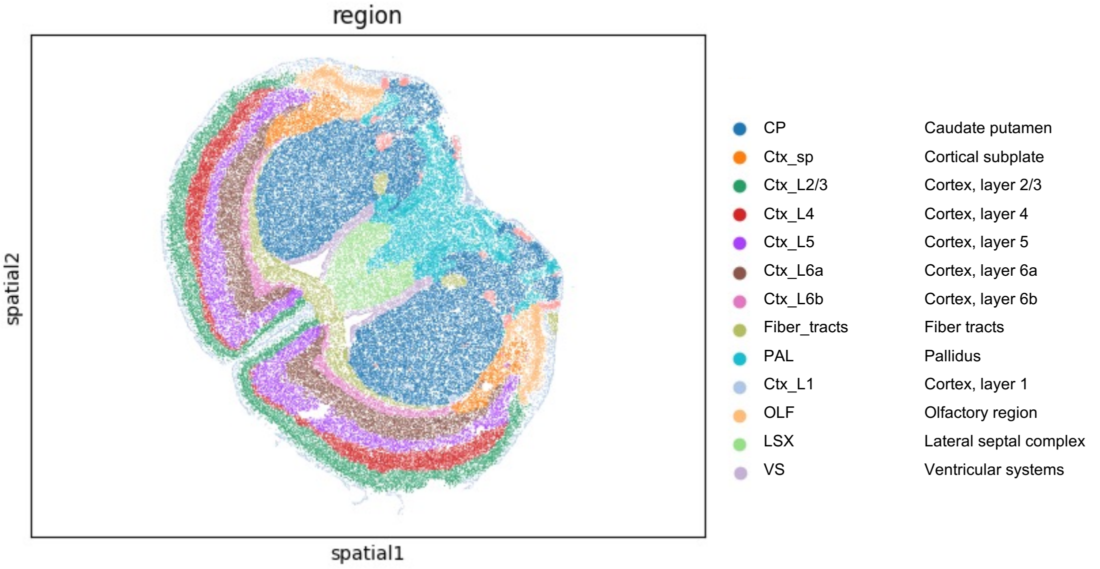

B

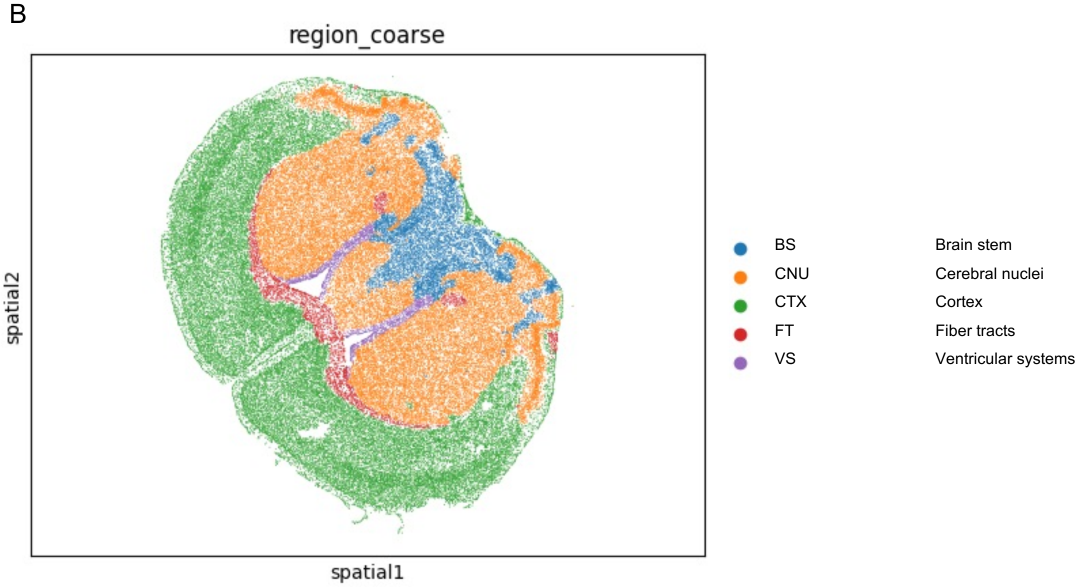

Supplementary Figure 37. Different resolution of MERSCOPE brain data
A: When using the default clustering resolution, MENDER successfully identifies fine brain structures, including different cortex layers (CTX L1-L6), Caudate putamen (CP), Cortical subplate (Ctx_sp), Olfactory region (OLF), Pallidus (PAL), Fiber tracts (Fiber_tracts), Ventricular systems (VS), and Lateral septal complex (LSX).
B: When we set the expected number of regions to 5 using the "res_search" function, MENDER accurately identifies 5 brain regions, including BS (Brain stem), CNU (Cerebral nuclei), CTX (Cortex), FT (Fiber tracts), and VS (Ventricular systems), aligning with the major brain regions defined in the Allen Brain Atlas.

Supplementary Figure 38

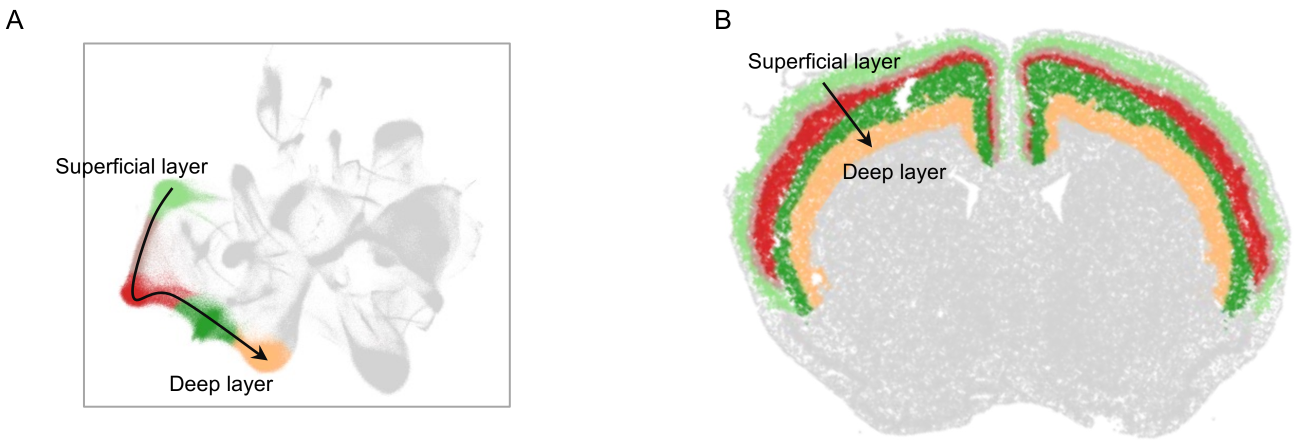

Supplementary Figure 38. MENDER UMAP of the whole MERSCOPE dataset (cortical region highlighted).
The MENDER UMAP of the whole dataset is shown in Fig. 4H, and the cortex layers are highlighted in (A). When mapping these clusters to the biological tissue space, one can see the the order in MENDER UMAP aligns well with the biological order of cortex layers (B).

Computing memory usage (MiB)							
Dataset	# Cells	STAGATE	BASS	SOTIP	SingleRange	CNC	MENDER
STARmap	3,190	2,149	1,236	2,865	755	1,027	873
BaristaSeq	11,426	3,663	1,568	3,462	921	1,352	956
MERFISH	378,918	82,766	N/A	N/A	38,655	26,798	56,537
MERSCOPE	734,696	N/A	N/A	N/A	53,541	N/A	86,635

Supplementary Table 1: Computing memory usage of all methods. For each method on each dataset, peak memory usage is recorded. The computing memory usage were examined for all the real data applications.

Note 1: Extended analysis on cell type identification task

We formulated four analyses, each contingent upon the availability of supervision signals and the nature of the annotation used.

- Analysis 1: MENDER is performed for unsupervised spatial domain identifications, the performance is quantified using NMI between identified domains and domain annotation. See the first row of Supplementary Figure 15.

- Analysis 2: MENDER is performed for unsupervised spatial domain identifications, the performance is quantified using NMI between identified domains and cell type annotation. See the second row of Supplementary Figure 15.

- Analysis 3: MENDER is performed for supervised spatial domain predictions, the performance is quantified using the prediction accuracy between predicted domain labels and domain annotation. See the third row of Supplementary Figure 15.

- Analysis 4: MENDER is performed for supervised cell type predictions, the performance is quantified using the prediction accuracy between predicted cell types and cell type annotation. See the fourth row of Supplementary Figure 15.

As MENDER's primary objective is unsupervised spatial domain identification, Analysis 1 has been thoroughly evaluated in the manuscript. Additionally, Analysis 3 was assessed in the original manuscript to measure MENDER's predictive capacity towards spatial domain annotations utilizing its context-aware representation. Although cell type identification is a separate task from spatial domain identification and isn't the central focus of this work, we have conducted additional analyses (Analyses 2 and 4) to explore MENDER's performance outside its designed scope, potentially providing motivation for other researchers in this field.

Analysis 2
Cell type annotations have much more mixing of labels compared with layer annotation. To evaluate MENDER's NMI by comparing the MENDER-identified spatial domains against the cell type annotations. We searched the spatial omics database (SODB) https://gene.ai.tencent.com/SpatialOmics/, and found two spatial transcriptomics dataset (i.e., osmFISH data and MERFISH data) that have both cell type (Supplementary Fig. 16B, 17B) and spatial domain annotations (Supplementary Fig. 16A, 17A).

For the osmFISH data, we first performed MENDER for unsupervised spatial clustering (Supplementary Fig. 16E), then evaluating the inferred spatial domain result using NMI, against the Domain annotation and Cell Type annotation, respectively. We next analyzed and compared between MENDER's result and the Cell type annotation. Visually, MENDER's prediction has clear layer pattern as low mixing and is more similar than the

Domain annotation, than the Cell type annotation. Quantitatively, the NMI between MENDER-identified domain and the Domain annotation is 0.743, substantially larger than the NMI between MENDER-identified domain and the Cell type annotation, i.e., 0.324 (Supplementary Fig. 16F). As such, both the visual and quantitative analysis indicated that MENDER's performance has reduction when analyzed against the Cell type annotation instead of the Domain annotation.

For the MERFISH data, similar conclusion can be drawn (Supplementary Fig. 17). The NMI between MENDER-identified domain (Supplementary Fig. 17E) and the Domain annotation (Supplementary Fig. 17A) is 0.634, substantially larger than the NMI between MENDER-identified domain (Supplementary Fig. 17E) and the Cell type annotation (Supplementary Fig. 17B), i.e., 0.109.

We then proceeded to test whether the conclusion holds true for other state-of-the-art spatial clustering methods. Specifically, we evaluated two recently published methods after 2022, namely STAGATE and SpaceFlow. When applied to the osmFISH data, both STAGATE (Supplementary Fig. 16C) and SpaceFlow (Supplementary Fig. 16D) exhibited spatial domains that visually resembled the domain annotation more than the cell type annotation. This finding was further supported by the quantitative NMI analysis (Supplementary Fig. 16F). Similar observations regarding SpaceFlow and STAGATE were made when analyzing the MERFISH data (Supplementary Fig. 17).

Analysis 4

For Analysis 4, we assembled nine spatial datasets from the SODB that contained Cell Type annotations. These datasets included five MERFISH data (from Ref[1]), three STARmap data (from Ref[2]), and one osmFISH data (from Ref[3]). As illustrated in Supplementary Fig. 15 fourth row (Analysis 4), we applied supervised classifiers on the context-aware representation generated by MENDER, using the cell type annotation as the supervision signal for each cell. The classification accuracy (sklearn.metrics.accuracy_score implementation) was reported as the median value from 5-fold cross-validation. The classifiers included Linear SVM (linearSVM), RBF SVM (rbfSVM), and Random Forest (RF). The prediction performance towards cell types using MENDER's representation was generally low, with accuracy centered around 0.5 (Supplementary Fig. 18).

We proceeded to test whether the representation generated by other state-of-the-art methods resulted in similar outcomes. We tested two recent methods, STAGATE and SpaceFlow, and found their performances (Supplementary Fig. 18) to be comparable, suggesting that current spatial domain identification methods are also not well suited to cell type identification tasks.

Note 2: Relationships and differences with Space-GM and CNC.

1 We have conducted a comprehensive analysis to distinguish MENDER's relationships and differences from Nolan's and Zou's work.

1.1 Nolan's work[4] (Cellular Neighborhood Clustering, CNC)

CNC is a groundbreaking work that integrates cellular context into spatial clustering. As per CNC's paper[4] and the corresponding GitHub page [https://github.com/nolanlab/NeighborhoodCoordination/blob/master/Neighborhoods/Neighborhood%20Identification.ipynb], CNC initially conducts cell clustering based on protein profiles from the CODEX data (in the case of spatial transcriptomics data, this could be substituted with gene expression profiles). Subsequently, in the tissue space, CNC identifies each cell's ten nearest neighbors (termed as "Cellular Neighborhood"), representing the central cell using the frequency of cell types (defined earlier) within its cellular neighborhood. Finally, the MiniBatch K-Means implementation of scikit-learn is applied to the representation. Therefore, the input for CNC is spatial omics data, which includes a gene expression matrix and a spatial coordination matrix; the output is the unsupervised identification of spatial domain labels for each cell.

1.2 Zou's work[5] (Space-GM).

Space-GM, a landmark work, employs a Graph Neural Network (GNN) model to predict patient outcomes (or other patient-level attributes) and identify disease-associated spatial motifs on multiplexed imaging data. According to Space-GM's paper[5] and the related GitHub page [https://gitlab.com/enable-medicine-public/space-gm/-/tree/main/], Space-GM first constructs a 3-hop neighborhood to encode the spatial relationships among cells, which is input into a GNN. The node embedding is pretrained by predicting the central cell's protein expression features and then fine-tuned for patient-level attribute prediction. The fine-tuned network generates the micro-environment embedding (similar concept to the embedding in STAGATE[6], SpaceFlow[7], and other deep learning-based spatial clustering methods. However, the difference is that the embedding of Space-GM is generated with supervision, unlike spatial clustering methods that are unsupervised). This can be used for spatial clustering. Although Space-GM is demonstrated with multiplex imaging data in the original paper, it can fundamentally be extended to spatial transcriptomics data with certain modifications, as discussed in the paper's discussion section. In summary, the input for Space-GM includes (1) different patients' spatial omics data, each containing a protein expression matrix and a spatial coordination matrix, and (2) patient-level attributes, e.g., primary outcome, survival length, recurrence, as shown in Figure 1b of Ref[5]. The output of Space-GM consists of patient-level attribute predictions and disease-associated motifs.

1.3 Relationships and differences between MENDER, CNC, and Space-GM

We next discuss the relationships between MENDER, CNC, Space-GM, and other popular spatial methods, in terms of Methodology, Supervision, and Task.

1.3.1 Methodology

Both MENDER and CNC construct the context-aware representation in a deterministic manner, while Space-GM (along with STAGATE, SpaceFlow, SpaGCN, and many others) creates the context-aware representation in a stochastic manner. By "stochastic", we refer to the fact that these methods require training and updating the context-aware representations and other parameters towards some loss functions, through stochastic optimization. "Deterministic", on the other hand, indicates that these methods do not need iterative updates and optimization parameters to obtain the context-aware representations. Based on the discussions about MENDER and CNC, it shows they efficiently count the cell type frequency within certain areas around the central cell to generate the representation, which is deterministic once the "cell type" is defined using standard single-cell clustering methods. The difference between MENDER and CNC is that MENDER captures multi-range context information around the central cell, while CNC captures information from just one range. Although fundamentally different in methodology, one similarity between MENDER and Space-GM is that they both use multiple ranges of context information around the central cell: MENDER uses cell frequencies in the multi-range cellular neighborhood, and Space-GM uses multi-hop neighborhood information. This might be why MENDER shows significant improvement over the one-range CNC.

1.3.2 Supervision

Regarding the supervision signal, both MENDER and CNC are unsupervised methods for spatial clustering. In contrast, Space-GM requires patient-level attributes as supervision signals to predict new patient outcomes and to fine-tune the microenvironment embeddings.

1.3.3 Task

The tasks of MENDER and CNC are both cell-level unsupervised prediction, while Space-GM is engaged in patient-level supervised prediction. Specifically, MENDER and CNC aim to delineate tissue structures in an unsupervised manner by clustering cells based on both spatial information and gene expression information. This aligns with other methods like SpaGCN, STAGATE, SOTIP, etc. This is also the reason why the MENDER manuscript benchmarks and analyzes these related unsupervised spatial clustering methods.

On the other hand, Space-GM's task is different. It trains the GNN model based on patient-level annotations and uses this to predict new patients' outcomes. Consequently, the benchmark studies in Space-GM's paper are against other patient-level prediction models.

Note 3: How MENDER's design contributes its performance

Based on our understanding and various recent review articles, modern state-of-the-art methods for modeling spatial omics data predominantly rely on encoding the spatial relationships of cells using a graph data structure, subsequently applying different operations to process and extract information from the graph[8-10]. As discussed above, "stochastic" methods require iteratively access and updates to the entire graph as well as network parameters. These methods are both computationally and memory intensive, especially those not designed for running on a GPU, such as SpatialPCA, BayesSpace, and BASS. Although these are all exceptional methods, they share a common issue of lengthy processing time. For example, refer to page 11 of the BayesSpace paper's supplementary file for BayesSpace's processing time (26.8 minutes for data of 10^3 cells), page 36 of the BASS paper's supplementary file for BASS's processing time (~10 minutes for data of 10^3 cells), and page 53 of the SpatialPCA paper's supplementary file for SpatialPCA's processing time (6 minutes for 10^3 cells). "Stochastic" methods that can run on a GPU mitigate the processing time issue to some extent, thanks to GPU parallelization. For instance, methods like SpaGCN and STAGATE can reduce the processing time for a dataset of 10^3 cells to 1-2 minutes. However, the problem of memory intensity for large datasets remains, particularly as GPU memory is far more expensive than CPU memory. This issue is evident when applying STAGATE to a MERFISH dataset containing 3×10^5 cells (the dataset is from Ref[11]) using a current state-of-the-art GPU (NVIDIA A100(80G)), which results in a memory error, as our following screenshot.

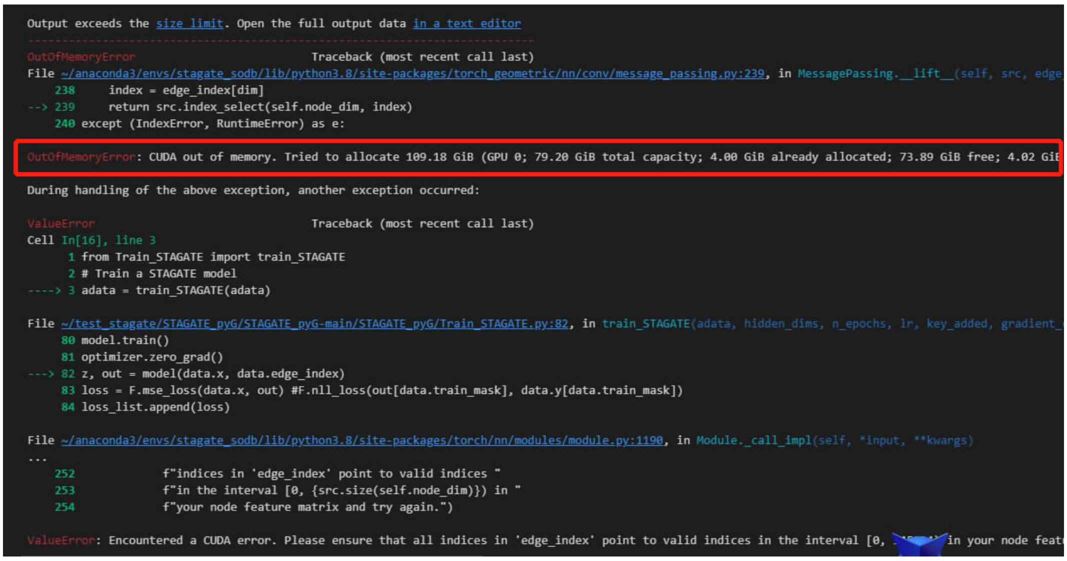

"Deterministic" methods such as CNC (Cellular Neighborhood Clustering, Nolan's Cell 2020 paper)[4] only need to store a sparse affinity matrix for retrieving the KNN spatial neighbors of each cell. This matrix is accessed only once to obtain the context-aware representation of cells. Additionally, "deterministic" methods do not require learnable parameters and embeddings to be stored, updated, and optimized, leading to improved running time and memory efficiency. However, although CNC is fast and memory-efficient, it only captures one range of the local neighborhood of each cell (KNN spatial graph), and its spatial clustering performance is not as proficient as the "stochastic" methods that

model multi-hop local spatial relationships.

We arrive at the conclusion that: (1) "Stochastic" methods are accurate but require more running time and memory, leading to scalability issues; (2) The available "deterministic" method (i.e., CNC) offers running time and memory efficiency, but its accuracy is relatively lower due to insufficient neighborhood modeling. MENDER seeks to retain the advantages of both paradigms. From the start, we can jointly conceive how MENDER should be designed to achieve (1) running time efficiency, (2) memory efficiency, and (3) effective neighborhood modeling.

To circumvent the running time issue, the design of MENDER has two options: (1) It must not have iterative optimization procedures, or (2) It should have an optimization procedure, but it needs to be parallelized by a GPU. To avoid the memory issue, the design of MENDER has to forgo the second option, since it needs to store both the spatial data graph per se and the network parameters to be trained, causing substantial memory usage in a GPU (running on a 3×10^5 dataset exceeds the capacity of an A100 GPU, as demonstrated earlier in this discussion). To enhance the neighborhood modeling capability, an approach should be designed to retain more information about the cellular spatial neighborhood than CNC does.

MENDER is designed following the above line of thought. Based on the consensus neighborhood structure, MENDER constructs a multi-range neighborhood to encode more information in its context-aware representation. MENDER's performance has been tested and found to be superior to the current state-of-the-art in terms of prediction accuracy, running time, and scalability to very large datasets. In addition, as discussed in the "Technical details" at the start of this response, we implemented additional software engineering to further parallelize MENDER's implementation, significantly enhancing the running time efficiency, especially when dealing with a high number of slices, and all without the need for a GPU.

Note 4: Evaluation of MENDER performance under various parameter settings.
As depicted in the schematic figure of MENDER (Figure 1), it has two tunable parameters accessible to the users: the number of ranges (# Ranges) and the size of each range (Radius).

Although we followed a consistent parameter setting, it is not to imply that slight deviations from these settings would result in significant variations in the methods' performance. To provide readers with a comprehensive view of the impact of parameter changes on MENDER's performance, we examined the effect of various parameter choices on 10 spatial transcriptomics datasets, as shown in Supplementary Fig. 9. These datasets span from single-cell resolution to non-single cell resolution data. For the #Ranges, we evaluated settings ranging from 1 to 10, incrementing by 1. For the Radius, we assessed settings ranging from 5μm to 50μm, in steps of 5μm.

Quantitatively, based on results from 3 datasets comprising 37 slices, we discerned that the performance of MENDER in terms of Normalized Mutual Information (NMI) remains relatively high for a particular range of #Ranges and Radius settings in most datasets (refer to the heatmaps in Supplementary Fig. 19, where deeper colors of red denote better performance). The heatmaps for each dataset primarily show higher NMI values in proximity to their counter diagonals. The settings we recommend (i.e., #Ranges=6, Radius=15μm) are within this high-performance range. This is further substantiated when analyzing each dataset jointly, as shown in Supplementary Fig. 20.

We also visualized how MENDER-identified tissue structures change under varying parameter settings, including datasets of single-cell resolution such as Stereo-seq (Supplementary Fig. 21), osmFISH (Supplementary Fig. 22), and STARmapPLUS (Supplementary Fig. 23-30), near single-cell resolution such as Slide-seq (Supplementary Fig. 32-34), and non-single-cell resolution data such as ST and 10x Visium (Supplementary Fig. 31). For single-cell-resolution data, taking one of the STARmapPLUS datasets as an example, which showcased a tissue containing the mouse cortex and hippocampus region, revealed some insights (Supplementary Fig. 24). High #Ranges combined with low Radius led to MENDER capturing multiple ranges of cellular context but having inadequate diversity within each range (e.g., Supplementary Fig. 24, blue box). On the other hand, high Radius paired with a low #Ranges meant that even if the single range had sufficient cell type diversity, focusing on just one range resulted in suboptimal performance, underscoring the importance of MENDER's multi-range features (e.g., Supplementary Fig. 24, red box). Furthermore, simultaneous high #Ranges and Radius can also induce challenges, such as over-smoothing, evident when identifying finer structures, where a part of CA3 (see Supplementary Fig. 13 for brain reference) was mis-identified (Supplementary Fig. 24, green box and black circle). For non-single-cell resolution data, due to fixed spatial spot layouts (for example, one spot of ST has 4 neighbors), only the #Ranges parameter is tunable. Here, we found that both extremely low and high #Ranges yielded unsatisfactory results, whereas a moderate

#Ranges (especially at #Ranges=3) produced best results, as showcased in Supplementary Fig. 31.

In summary, we offer parameter setting recommendations for various spatial technologies. With these settings, good results can be expected. Users can also customize these parameters as per their specific requirements. Our experiments suggest that the outcomes of MENDER gradually vary with parameter settings rather than experiencing abrupt changes. While extreme settings might result in undesirable results, MENDER typically yields reasonable results within certain parameter ranges.

References

1 Moffitt, J. R. *et al.* Molecular, spatial, and functional single-cell profiling of the hypothalamic preoptic region. *Science* **362**, doi:10.1126/science.aau5324 (2018).

2 Wang, X. *et al.* Three-dimensional intact-tissue sequencing of single-cell transcriptional states. *Science* **361**, doi:10.1126/science.aat5691 (2018).

3 Codeluppi, S. *et al.* Spatial organization of the somatosensory cortex revealed by osmFISH. *Nat. Methods* **15**, 932-+, doi:10.1038/s41592-018-0175-z (2018).

4 Schürch, C. M. *et al.* Coordinated Cellular Neighborhoods Orchestrate Antitumoral Immunity at the Colorectal Cancer Invasive Front. *Cell*, doi:10.1016/j.cell.2020.07.005 (2020).

5 Wu, Z. *et al.* Graph deep learning for the characterization of tumour microenvironments from spatial protein profiles in tissue specimens. *Nat Biomed Eng*, doi:10.1038/s41551-022-00951-w (2022).

6 Dong, K. & Zhang, S. Deciphering spatial domains from spatially resolved transcriptomics with an adaptive graph attention auto-encoder. *Nat Commun* **13**, 1739, doi:10.1038/s41467-022-29439-6 (2022).

7 Ren, H., Walker, B. L., Cang, Z. & Nie, Q. Identifying multicellular spatiotemporal organization of cells with SpaceFlow. *Nat Commun* **13**, 4076, doi:10.1038/s41467-022-31739-w (2022).

8 Vandereyken, K., Sifrim, A., Thienpont, B. & Voet, T. Methods and applications for single-cell and spatial multi-omics. *Nat Rev Genet*, 1-22, doi:10.1038/s41576-023-00580-2 (2023).

9 Zeng, Z., Li, Y., Li, Y. & Luo, Y. Statistical and machine learning methods for spatially resolved transcriptomics data analysis. *Genome Biol* **23**, 83, doi:10.1186/s13059-022-02653-7 (2022).

10 Palla, G., Fischer, D. S., Regev, A. & Theis, F. J. Spatial components of molecular tissue biology. *Nat Biotechnol*, doi:10.1038/s41587-021-01182-1 (2022).

11 Allen, W. E., Blosser, T. R., Sullivan, Z. A., Dulac, C. & Zhuang, X. Molecular and spatial signatures of mouse brain aging at single-cell resolution. *Cell*, doi:10.1016/j.cell.2022.12.010 (2022).
