# Supplementary material for: MENDER: fast and scalable tissue structure identification in spatial omics data
Source: Nat Commun. 2024 Jan 5;15:207. doi: 10.1038/s41467-023-44367-9 (PMC10770058; doi:10.1038/s41467-023-44367-9)
Supplement: Supplementary file 3 — Reporting Summary [file 41467_2023_44367_MOESM3_ESM.pdf]

nature portfolio

Corresponding author(s): Zhiyuan Yuan

Last updated by author(s): Nov 10, 2023

Reporting Summary

Nature Portfolio wishes to improve the reproducibility of the work that we publish. This form provides structure for consistency and transparency in reporting. For further information on Nature Portfolio policies, see our Editorial Policies and the Editorial Policy Checklist.

Statistics

For all statistical analyses, confirm that the following items are present in the figure legend, table legend, main text, or Methods section.

n/a	Confirmed	
☐	☒	The exact sample size (*n*) for each experimental group/condition, given as a discrete number and unit of measurement
☐	☒	A statement on whether measurements were taken from distinct samples or whether the same sample was measured repeatedly
☐	☒	The statistical test(s) used AND whether they are one- or two-sided<br>*Only common tests should be described solely by name; describe more complex techniques in the Methods section.*
☐	☒	A description of all covariates tested
☐	☒	A description of any assumptions or corrections, such as tests of normality and adjustment for multiple comparisons
☐	☒	A full description of the statistical parameters including central tendency (e.g. means) or other basic estimates (e.g. regression coefficient) AND variation (e.g. standard deviation) or associated estimates of uncertainty (e.g. confidence intervals)
☐	☒	For null hypothesis testing, the test statistic (e.g. *F*, *t*, *r*) with confidence intervals, effect sizes, degrees of freedom and *P* value noted<br>*Give P values as exact values whenever suitable.*
☒	☐	For Bayesian analysis, information on the choice of priors and Markov chain Monte Carlo settings
☒	☐	For hierarchical and complex designs, identification of the appropriate level for tests and full reporting of outcomes
☒	☐	Estimates of effect sizes (e.g. Cohen's *d*, Pearson's *r*), indicating how they were calculated

Our web collection on statistics for biologists contains articles on many of the points above.

Software and code

Policy information about availability of computer code

Data collection	Raw data are collected using Python3.9.
Data analysis	scanpy==1.9.1 squidpy==1.2.3 numpy==1.22.4 pandas==1.5.1 scipy==1.9.3 matplotlib==3.6.0 seaborn==0.12.1 palettable==3.3.0 scikit-learn==1.1.2 scikit-image==0.19.3<br>Opykernel==6.16.1<br>Our custom software is available at https://github.com/yuanzhiyuan/SOMENDER.

For manuscripts utilizing custom algorithms or software that are central to the research but not yet described in published literature, software must be made available to editors and reviewers. We strongly encourage code deposition in a community repository (e.g. GitHub). See the Nature Portfolio guidelines for submitting code & software for further information.

Data

Policy information about availability of data

All manuscripts must include a data availability statement. This statement should provide the following information, where applicable:

- Accession codes, unique identifiers, or web links for publicly available datasets
- A description of any restrictions on data availability
- For clinical datasets or third party data, please ensure that the statement adheres to our policy

STARmap Prelimbic area data: [https://www.starmapresources.com/data];

BaristaSeq Visual cortex data: [https://download.brainimagelibrary.org/06/35/0635a0];
MERFISH Frontal cortex data: [https://cellxgene.cziscience.com/collections/ 31937775-0602-4e52-a799-b6acdd2bac2e];
MERSCOPE (Vizgen) Brain data: [https://info.MERSCOPE.com/mouse-brain-data], it can also be downloaded from SODB [https://gene.ai.tencent.com/SpatialOmics/dataset?datasetID=152];
ST Olfactory bulb data: [https://www.spatialresearch.org/resources-published-datasets/];
Visium data: [https://www.10xgenomics.com/resources/datasets];
Slide-seq data: [https://singlecell.broadinstitute.org/single_cell/study/SCP815/sensitive-spatial-genome-wide-expression-profiling-at-cellular-resolution#study-summary];
Stereo-Seq data: [https://db.cngb.org/stomics/datasets/STDS0000058];
osmFISH data: [http://linnarssonlab.org/osmFISH/];
ExSeq data: [10.5281/zenodo.4075515];
STARmapPLUS data: [https://singlecell.broadinstitute.org/single_cell/study/SCP1375];
Allen Reference Atlas: [https://mouse.brain-map.org/experiment/thumbnails/100048576?image_type=atlas];
We also provide the benchmark datasets as h5ad format via SODB, please find how to load them in the tutorial [https://mender-tutorial.readthedocs.io/en/latest/].

Research involving human participants, their data, or biological material

Policy information about studies with human participants or human data. See also policy information about sex, gender (identity/presentation), and sexual orientation and race, ethnicity and racism.

Reporting on sex and gender	Not relevant.
Reporting on race, ethnicity, or other socially relevant groupings	Not relevant.
Population characteristics	Not relevant.
Recruitment	Not relevant.
Ethics oversight	Not relevant.

Note that full information on the approval of the study protocol must also be provided in the manuscript.

Field-specific reporting

Please select the one below that is the best fit for your research. If you are not sure, read the appropriate sections before making your selection.

☒ Life sciences ☐ Behavioural & social sciences ☐ Ecological, evolutionary & environmental sciences

For a reference copy of the document with all sections, see nature.com/documents/nr-reporting-summary-flat.pdf

Life sciences study design

All studies must disclose on these points even when the disclosure is negative.

Sample size	There are no in-house data in this study, and all the data used in this study are from public papers. So no sample size calculation was performed. The rationale for these sample sizes are besed on related methods papers published earlier, and we included more sample datasets to benchmark our method than related method papers.
Data exclusions	None data were excluded from the raw.
Replication	Each experiments were replicated for 5 runs.
Randomization	Randomization was achieved by setting random seeds.
Blinding	The algorithm developer and the data analyzer are the same person, so totally blinding is impossible.

Reporting for specific materials, systems and methods

We require information from authors about some types of materials, experimental systems and methods used in many studies. Here, indicate whether each material, system or method listed is relevant to your study. If you are not sure if a list item applies to your research, read the appropriate section before selecting a response.

Materials & experimental systems

n/a	Involved in the study	
☒	☐	Antibodies
☒	☐	Eukaryotic cell lines
☒	☐	Palaeontology and archaeology
☒	☐	Animals and other organisms
☒	☐	Clinical data
☒	☐	Dual use research of concern
☒	☐	Plants

Methods

n/a	Involved in the study	
☒	☐	ChIP-seq
☒	☐	Flow cytometry
☒	☐	MRI-based neuroimaging

Plants

Seed stocks	Not relevant.
Novel plant genotypes	Not relevant.
Authentication	Not relevant.
